# Chromatin interactions and candidate genes at ten prostate cancer risk loci

**DOI:** 10.1038/srep23202

**Published:** 2016-03-16

**Authors:** Meijun Du, Lori Tillmans, Jianzhong Gao, Ping Gao, Tiezheng Yuan, Rachel L Dittmar, Wei Song, Yuehong Yang, Natasha Sahr, Tao Wang, Gong-Hong Wei, Stephen N. Thibodeau, Liang Wang

**Affiliations:** 1 Department of Pathology, MCW Cancer Center, Medical College of Wisconsin, Milwaukee, 53226, WI, USA; 2 Department of Laboratory Medicine and Pathology, Mayo Clinic, Rochester, 55905, MN, USA; 3 Beijing 3H Medical Technology Co. Ltd., Beijing, 100176, China; 4 Division of Biostatistics, Institute for Health & Society, Medical College of Wisconsin, Milwaukee, 53226, WI, USA; 5 Faculty of Biochemistry and Molecular Medicine and Biocenter Oulu, University of Oulu, Oulu, Finland

## Abstract

Genome-wide association studies have identified more than 100 common single nucleotide polymorphisms (SNPs) that are associated with prostate cancer risk. However, the vast majority of these SNPs lie in noncoding regions of the genome. To test whether these risk SNPs regulate their target genes through long-range chromatin interactions, we applied capture-based 3C sequencing technology to investigate possible *cis*-interactions at ten prostate cancer risk loci in six cell lines. We identified significant physical interactions between risk regions and their potential target genes including *CAPG* at 2p11.2, *C2orf43* at 2p24.1, *RFX6* at 6q22.1, *NFASC* at 1q32.1, *MYC* at 8q24.1 and *AGAP7P* at 10q11.23. Most of the interaction peaks were co-localized to regions of active histone modification and transcription factor binding sites. Expression quantitative trait locus (eQTL) analysis showed suggestive eQTL signals at rs1446669, rs699664 and rs1078004 for *CAPG* (p < 0.004), rs13394027 for *C2orf43* (p = 2.25E-27), rs10993994 and rs4631830 for *AGAP7P* (p < 8.02E-5). Further analysis revealed an enhancer activity at genomic region surrounding rs4631830 which was expected to disrupt HOXB-like DNA binding affinity. This study identifies a set of candidate genes and their potential regulatory variants, and provides additional evidence showing the role of long-range chromatin interactions in prostate cancer etiology.

Genome-wide association studies (GWAS) have identified thousands of single-nucleotide polymorphisms (SNPs) that are associated with various diseases^1^,^2^,^3^. For prostate cancer, more than 100 loci have been reported to contain genetic risk variants^4^,^5^,^6^,^7^,^8^,^9^,^10^,^11^. However, functional characterization of causal variants and their target genes still remains a challenge. In many risk loci, the genetic variants reported in GWAS are not necessarily causative themselves, but are in strong linkage disequilibrium (LD) with the true causal variants as part of large haplotypes^4^,^5^,^6^,^7^,^8^,^9^,^10^,^11^. To date, the vast majority of these reported risk variants have been mapped to intergenic or intronic regions of the genome and many of these lie some distance from the nearest protein-coding genes^12^,^13^,^14^. This observation implies that these risk SNPs or their LD SNPs may reside in regulatory elements, executing their effects through long-range regulation of gene expression, rather than by directly affecting gene function^15^,^16^,^17^,^18^.

Chromosome Conformation Capture (3C)^19^ and its derivatives have become an invaluable tool for functional annotation of noncoding sequence variants^18^,^20^, and are used to link SNPs in regulatory elements to their target genes^21^,^22^,^23^. Recently, capture-based 3C (Capture 3C-seq) technologies such as Capture-C, 3C-MTS, and Capture Hi-C have been published by independent laboratories and were able to analyze hundreds of cis-interactions at high resolution in a single high-throughput experiment^24^,^25^,^26^,^27^,^28^. These technologies allow high-resolution survey of the whole genome for potential interactions with multiple regions of interest simultaneously. For a given GWAS risk locus where the causal SNP and target genes are often unknown, it is necessary to examine the entire risk LD block at high resolution for its targets across the genome. Here we applied capture-based 3C-seq^26^ to identify potential cis-regulated genes that physically interacted with predicted regulatory elements at 10 prostate cancer risk loci. Our results demonstrate that the 3C-seq along with expression quantitative trait loci (eQTL) and other epigenetic analyses will facilitate identification of functional variants and candidate genes responsible for prostate cancer risk.

## Results

### Prostate cancer risk loci and selected capture regions

To examine the possible interactions between risk SNP loci and candidate target genes, we designed a SureSelect custom target enrichment assay covering the ten risk loci across entire LD blocks. Supplementary Table S1 lists ten prostate cancer risk loci, risk SNPs, targeted region sizes, and number of EcoRI sites in each locus. The total target region covered 1 Mb containing 300 EcoRI restriction cut sites. Capture probes were 120-mer RNA baits tiling upstream and downstream 250 bp at each EcoRI site. The probes were named by the number of EcoRI sites from first cut (E1) to last cut (E*) (Supplementary Table S2) on each chromosome. Six cell lines were used including two prostate cancer cell lines (LNCaP and DU-145), two normal prostate epithelial cell lines (BPH-1 and RWPE-1) and two EBV (Epstein-Barr virus) transfected lymphoblastoid cell lines (LCL). The LNCaP cell line was repeated to evaluate the reproducibility of the capture-based 3C assay.

### 3C-MTS data and reproducibility

To map the sequencing data, we first trimmed adaptor and low quality sequences, and then submitted ∼200 million read pairs per library for sequence alignments. Mappable read pairs accounted for ∼82% of all submitted sequences. Among these, ∼23.4% (∼38.17 million read pairs/library) was mapped to the target regions. These on-target sequences were defined as paired sequence reads with at least one end mapped to ≤250 bp to the nearest *Eco*RI site at risk regions of interest. The on-target sequences were further grouped as cis- and trans-interactions, which contributed to 91.2% and 9.8% of interactions, respectively. The sequence mapping data for the six cell lines is summarized in Table 1. From these on-target sequences, we further excluded all duplicated reads (both ends had identical sequences) to avoid amplification bias during sequencing library preparation. The probe regions which were located in repeat sequences or had low capture efficiency were excluded. Finally, a total 466 probe regions remained for data analysis.

To evaluate the reproducibility of the 3C-MTS libraries, we tested the two independent data sets from the LNCaP cell line. We applied Pearson correlation coefficient analysis in the dataset with at least one read in both data sets and observed significant correlation (*r* = 0.90) between original and the repeat library. This correlation was further enhanced if considering the dataset with at least three read counts in both data sets (*r* = 0.93). Meanwhile, we also observed significant correlation between the LNCaP and other cell lines tested (Supplementary Table S3).

### Cis-chromatin interactions and epigenetic ChIP-seq analysis at risk loci

To determine the significant interactions at each probe site, we applied fourSig software program^29^ by summarizing the total number of reads in each window to generate an observed distribution. The program then created a randomized distribution by shuffling the observed reads among mappable 3C fragments and calculating a significance threshold from the top fifth percentile of 1000 calculations. The window size was defined as 1 and the threshold of the false discovery rate (FDR) was defined as 0.01. The most common and significant interactions for the ten regions in 6 cell lines are listed in Table 2. Overall, we observed the most significant interactions within +/−1 Mb around the target regions. Some of these tended to be more specific interaction between capture fragments and one target gene, such as 2p11.2 locus for *CAPG*. Some demonstrated multiple interactions between separate fragments at one risk locus and different target genes, such as 6q22.1 for *RFX6* and *GPRC6A*. Meanwhile, we observed tissue-specific and cell line-specific interactions, such as 1q32.1 for *NFASC* and 8q24 for *MYC*.

### 2p11.2 risk locus

**Figure 1:**
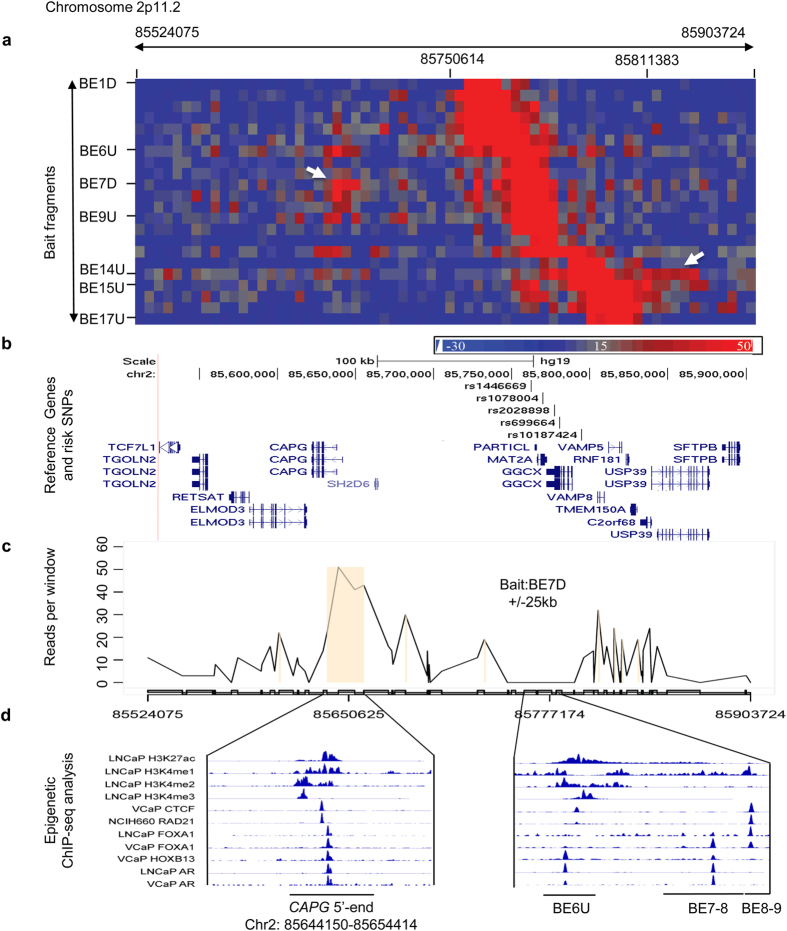
Chromatin interactions and functional annotation at 2p11.2. (**a**) Physical interaction heatmap. Y-axis lists 17 EcoRI sites where 22 bait fragments are shown from BE1 (chr2: 85750614) to BE17 (chr2: 85811383). X-axis is EcoRI-defined fragments from chr2: 85524075 to chr2: 85903724 (66 EcoRI sites). Increased signals between chr2: 85750614 and 851811383 are self or near ligations. White arrows indicate high interaction hot spots. (**b**) Risk SNPs and nearby genes in the same genomic region are shown in (**a**). (**c**) Chromatin interactions between bait BE7D and EcoRI fragments from chr2: 85524075 to 85903724. The significant interactions are between BE7D and the 5′-end of *CAPG.* Statistically significant interactions are highlighted in light brown. Signals at the bait and +/−25 kb regions are excluded due to high levels of self or nearby ligations. (**d**) Epigenomic marks at the interaction hot spots.

The association of this locus with prostate cancer has been previously reported^30^,^31^. rs2028898 was nominally associated with prostate cancer risk in Japanese population studies^30^. rs10187424 was reported as a prostate cancer susceptibility locus in a GWAS, in particular, in cases with a family history of prostate cancer^31^. The LD block covering the risk locus spans a 73 kb genomic region with 17 EcoRI cut sites. 22 probe bait regions were used for final data analysis, designated as from BE1U, BE1D to BE17U. The 3C-seq data showed significant and specific interactions between bait region BE6-9 (chr2: 85768683-85778503) and several consecutive EcoRI fragments (chr2: 85636964–85660165) at the 5′-end region of *CAPG* (Fig. 1a,b). The most significant interaction was between BE7D (Fig. 1c) and 5′-end of *CAPG.* We also found strong interactions between BE9U, BE6U and the 5′-end of *CAPG*. These interactions were found in all tested cell lines (Supplementary Fig. S1, Table 2). Further ChIP-seq analysis in prostate cancer cell lines indicated multiple positive signals in the EcoRI fragment BE8-9 (chr2: 85778503–86781283) including H3K4me1 and FOXA1 in LNCaP; CTCF and FOXA1 in VCaP, and RAD21 in NCIH660. For region containing EcoRI fragment BE7-8 (bait BE7D), the positive epigenetic signals included H3K4me1, AR, and FOXA1 in LNCaP, AR, FOXA1 and HOXB13 in VCaP (Fig. 1d). For the *CAPG* region where bait frequently interacted, we observed multiple histone modifications and TF binding sites at EcoRI fragment chr2: 85644150–85654414. In addition, we observed the interactions between BE14-15 (chr2: 85804733–85805038) and 5′-end of *USP39* (chr2: 85841299–85871912) (Fig. 1, Table 2).

### 1q32.1 risk locus

**Figure 2:**
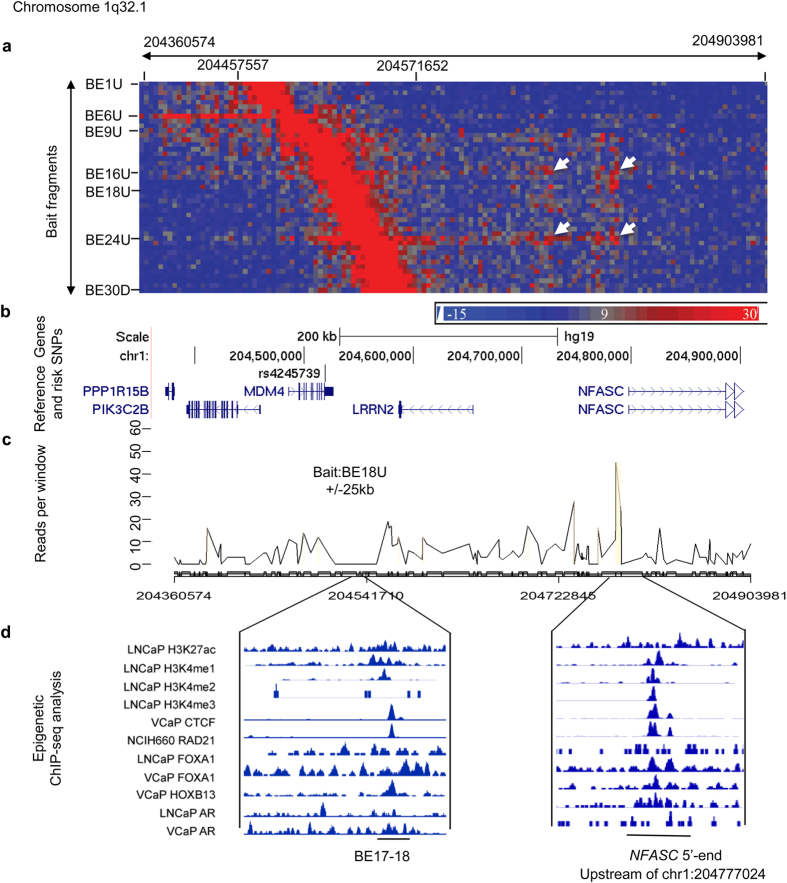
Chromatin interactions and functional annotation at 1q32.1. (**a**) Physical interaction heatmap. Y-axis lists 30 EcoRI sites where 46 bait fragments are shown from chr1: 204457557 to chr1: 204571652. X-axis is EcoRI-defined fragments from chr1: 204360574 to chr1: 204903981 (135 EcoRI sites). Increased signals between chr1: 204457557 and 204571652 are self or near ligations. White arrows indicate high interaction hot spots. (**b**) Risk SNPs and nearby genes in the same genomic region are shown in (**a**). (**c**) Chromatin interactions between bait BE18U and EcoRI fragments from chr1: 204360574 to chr1: 204903981. The significant interaction is between BE18U and the 5′-end of gene *NFASC.* Statistically significant interactions are highlighted in light brown. Signals at the bait and +/−25 kb regions are excluded due to high levels of self or nearby ligations. (**d**) Epigenomic marks at the interaction hot spots.

A previous study shows that rs4245739 at this locus is associated with prostate cancer^32^. Another SNP rs1380576 (LD with rs4245739, *r*^2^ = 0.89) has been linked to prostate cancer aggressiveness^33^. The LD block covering this risk locus spans a 120 kb genomic region with 30 EcoRI cut sites. After filtering, a total of 46 probe bait regions designated as from BE1U, BE1D to BE30D were used for the final data analysis. 3C-seq data showed that the significant interactions occurred outside right boundary of the bait region. Hot interaction spots were between two separate capture fragment clusters and two separate target regions close to the 5′-end of *NFASC* (Fig. 2a,b). One of the capture fragment clusters was between BE16 and BE18 (chr1: 204518514–204536408) across three EcoRI fragments with interaction peak at BE18U (chr1: 204536408) (Fig. 2c). The interaction peaks upstream of BE18 were accompanied with high H3K27ac, H3K4me1 and H3K4me2 signals. In addition, CTCF, RAD21, FOXA1, HOXB13 and AR binding sites were overlapped with the E18U fragment (Fig. 2d). Another cluster was between BE23 and BE25 (chr1: 204550984–204561889) across two EcoRI fragments with an interaction peak at BE24U (chr1: 204550984). For the target gene *NFASC* region, one of these interactions covered five EcoRI sites with a peak at chr1: 204777024. ChIP-seq data indicated a strong H3K4me3 signal in LNCaP cell line upstream of EcoRI site at chr1: 204777024, where a strong AR binding was also observed in the LNCaP cell line, CTCF, FOXA1, HOXB13 binding in VCaP cell line, and RAD21 binding in NCIH600 cell line (Fig. 2d). Another target gene region was found at the EcoRI fragment chr1: 204728019–204737511, 69.8 kb away from the gene *NFASC*. These interactions appeared to be tissue and cell-line specific, with stronger signals in prostate cell lines and weak or no signals in LCLs (Supplementary Fig. 2). The interaction hot spot outside of the left boundary of the target gene region covered chr1: 204375333–204383744 at the 5′-end of gene *PPP1R15B* and chr1: 204404188-204426713 at the intron of gene *PIK3C2B*. The interacting bait regions included BE6U (chr1: 204475811) and BE9-10 (chr1: 204482247–204489040) with various strengths of signals among different cell lines (Supplementary Fig. S2, Table 2).

### 10q11.23 risk locus

**Figure 3:**
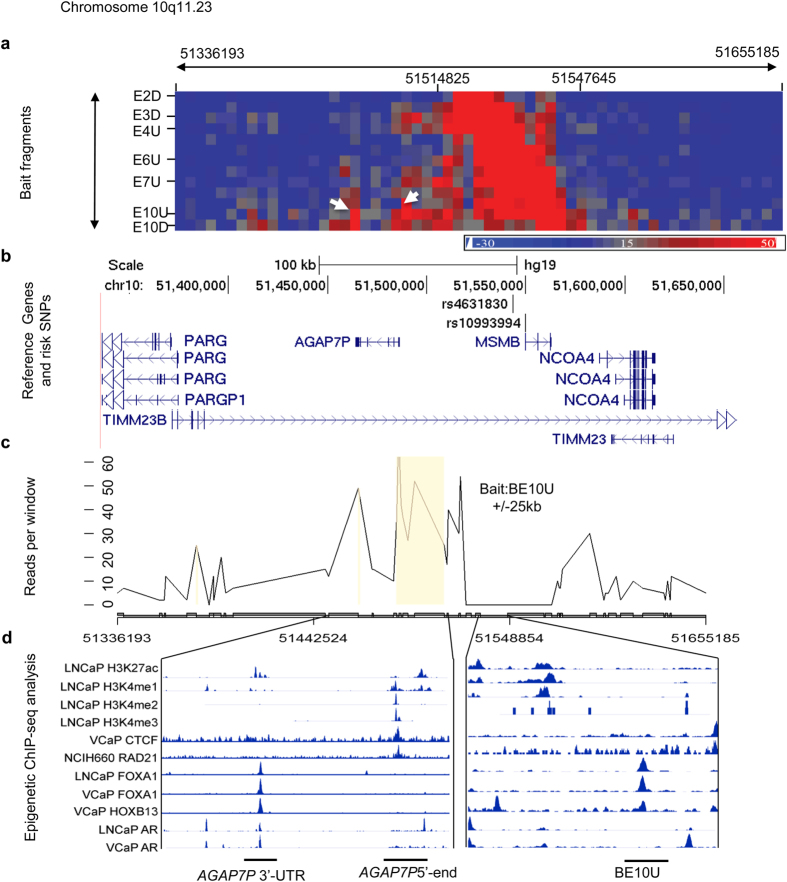
Chromatin interactions and functional annotation at 10q11.23. (**a**) Physical interaction heatmap. Y-axis lists 10 EcoRI sites where 13 bait fragments are shown from chr10: 51514825 to 51547645. X-axis is EcoRI-defined fragments from chr10: 51336193 to 51655185 (60 EcoRI sites). Increased signals between chr10: 51514825 and 51547645 are self or near ligations. White arrows indicate interaction hot spots. (**b**) Risk SNPs and nearby genes in the same genomic region are shown in (**a**). (**c**) Chromatin interactions between bait E10U and EcoRI fragments from chr10: 51336193 to 51655185. The significant interaction is between E10U and the 3′-and 5′-ends of *AGAP7P*. Statistically significant interactions are highlighted in light brown. Signals at the bait and +/−25 kb regions are excluded due to high levels of self or nearby ligations. (**d**) Epigenomic marks at the interaction hot spots.

Two risk SNPs (rs10993994 and rs4631830) at this locus were associated with an increased risk of developing prostate cancer^6^,^34^,^35^. The LD block covering the risk locus spans a 47 kb genomic region with 10 EcoRI cut sites. 13 probe bait regions designated as from E2D, E3U to E10D were used for final data analysis. 3C-seq analysis showed significant interactions between baits (E10U and E10D) and several EcoRI sites at the 5′-end of gene *AGAP7P* from chr10: 51474882 to chr10: 51497238 (Fig. 3a,b). The most significant interactions were between E10U and fragments covering the 5′-end of *AGAP7P*. The E10U was also interacted with 3′-UTR of *AGAP7P* at EcoRI site of chr10: 51466745 (Fig. 3c). ChIP-seq data showed transcription factors (FOXA1, HOXB13, AR, RAD21) binding at the 3′-end fragment and strong histone modification (H3K27ac, H3K4me1, H3K4me2 and H3K4me3) signal at the 5′-end fragment of the gene (Fig. 3d). For E10U fragment, we observed FOXA1 and HOXB13 binding signals. These interactions appeared to be tissue and cell-line specific, with stronger signals in prostate cell lines and weak or no signals in LCLs (Table 2, Supplementary Fig. S3)

### 8q24 risk locus

**Figure 4:**
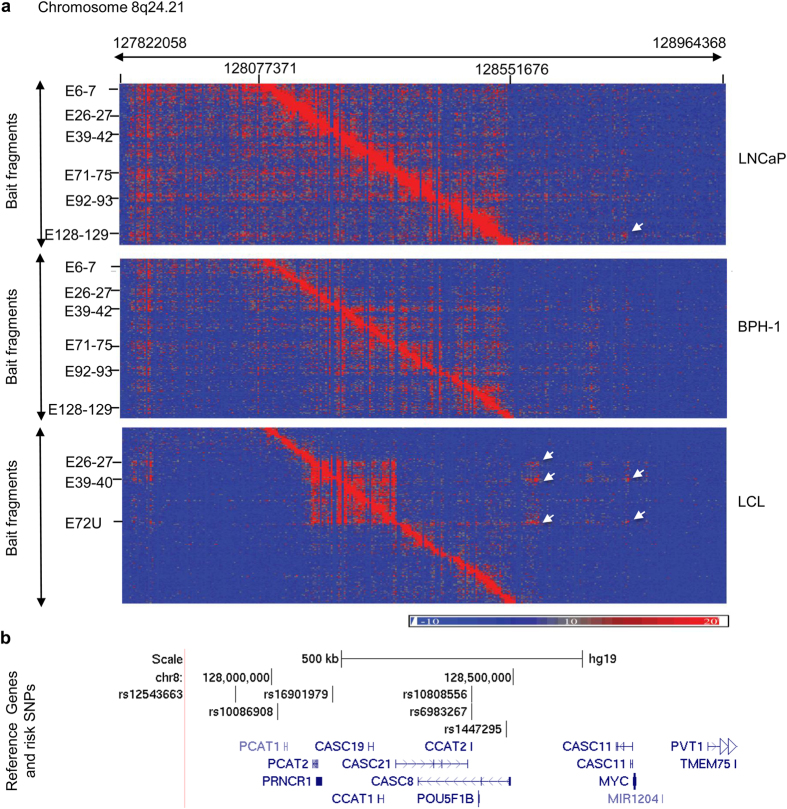
TAD structure and chromatin interaction at 8q24.21 risk locus. (**a**) Cell line-specific chromatin interacting domain at 8q24 risk locus in prostate cancer (LNCaP), normal (BPH-1) and LCL cell lines. Y-axis lists 135 EcoRI sites where 213 bait fragments are shown from E1 (chr8: 128077371) to E135 (chr8: 128551676). X-axis is EcoRI-defined fragments from chr8: 127822058 to 128964368 (325 EcoRI cutting sites). TAD structure is clearly shown in LCL but not in LNCaP cell line. The red signals in diagonal lines are self or nearby ligations. The white arrows indicate interaction hot spots. (**b**) Risk SNPs and nearby genes in the same genomic region shown in (**a**).

This risk locus has been extensively reported in previous studies^4^,^8^,^15^,^16^,^36^,^37^,^38^,^39^. 8q24 contains at least three genomic risk regions (region 1, region 2 and region 3), which contribute independently to prostate cancer risk^36^,^37^,^38^. This risk locus has 135 EcoRI cut sites with 213 probe-designable fragments. We observed novel topologically associating domain (TAD) structures from different cell types at this locus, in addition to common findings including the regulatory elements (E6-7 for risk region 2, E92-93 for risk region 3, E128-129 for risk region 1 and E72D, E75U for unannotated risk region), and their target genes *MYC* and *PVT1*^26^,^36^. For prostate cancer cell lines, there was no clear TAD boundary within bait regions (chr8: 128077371–129551676). Interactions within the region tended to be more random. However, for LCL cell lines, we observed a TAD with clear boundary between chr8: 12818750 and 12832217. Interactions were extremely high within the domain and low outside the domain (Fig. 4). Outside bait regions, cancer cell lines and LCLs also demonstrated different interaction patterns. Cancer cell lines showed more widespread but weak interactions while LCLs demonstrated clear hot spots at different genome positions. For example, we observed strong interactions of *MYC* gene region with prostate cancer risk region 1 (E128-129) in the LNCaP cancer cell line but not in LCLs. In contrast, strong interactions with the *MYC* region were at E72U and E39–40 in LCLs but not in prostate cancer cell lines. Additionally, LCL cell lines had three regions (E26-27, E39-40 and E71-75) showing unique interactions with a genomic region at chr8: 128565240–128578550 which covered the 5′-end of gene *CASC8 (LOC727677)*. As expected, the interaction pattern and intensity in normal prostate epithelial cell lines BPH-1 and RWPE-1 always remained between cancer cell lines and LCLs.

### Other target loci

In addition to the interactions listed above, we also observed significant interactions at other gene loci including *UBE2Q1* and *KCNN3* at 1q21.3, *HS1BP3*, C2orf43 at 2p24.1, *TRIP13* at 5p15.33, *RFX6, GPRC6A* at 6q22.1, *ANTXRLP1* at 10q11.23 and non-coding RNA LOC338694 at 11q13.2 (Table 2). Among these interactions, bait fragments AE3D (chr2: 20879971) and AE6D (chr2: 20853970) at 2p24.1 showed interactions with the 5′-end of *HS1BP3* (chr2: 20849923–20864079) and two separate regions at the 5′-end of gene *C2orf43* (chr2: 21111995–21122083 and chr2: 21130466–21184716). The interaction hot spot at fragment chr2: 21111995–21122083 was only observed in prostate cancer cell lines LNCaP and DU-145. Other interaction hot spots at multiple EcoRI fragments (chr2: 21130466–21184716) were observed in prostate-derived cell lines (LNCaP, DU-145, BPH-1 and RWPE-1), but not in LCLs. Bait fragment AE2D (chr5: 1275548) demonstrated an interaction with the 3′-end of gene *TRIP13* (chr5: 919694–928218) at 5p15.33. For the 10q11.23 locus, E7U and E9-10 showed interactions with the 5′-end of the gene *ANTXRLP1*. E4-5 and E12-13 exhibited physical contact with the non-coding RNA *LOC338694*. Multiple bait fragments AE5-7 (chr1: 154844039–154859292) and AE11-12 (chr1: 154888031–154904153) at 1q21.3 showed higher contact frequency with multiple locations of *KCNN3*.

### Associations of candidate risk SNPs and target genes expression

To determine whether the risk SNPs and their LD SNPs were associated with expression levels of target genes *CAPG, C2orf43, NFASC, RFX6, MYC, PVT1,* and *AGAP7P,* we performed eQTL analysis using RNA-seq and SNP genotyping data from 467 normal prostate tissues. We observed suggestive evidence of the SNP-gene expression associations at most loci tested (Supplementary Table S4). For examples, rs1446669 (a proxy for rs2028898, r^2^ = 1), rs699664 (a proxy for rs2028898, r^2^ = 0.90) and rs1078004 (a proxy for rs10187424, r^2^ = 0.94) were associated with the expression of *CAPG* (p value < 0.004). rs13394027 (a proxy for rs13385191, r^2^ = 0.89) was associated with the expression of *C2orf43* (p value = 2.25E-27). rs10808556 and rs6983267 (8q24.21) were associated with the expression of *PVT1* with p value = 8.38E-4 and 1.75E-3, respectively. rs10993994 and rs4631830 (10q11.23) were associated with the expression of *AGAP7P* with p value = 4.06E-07 and 8.02E-5, respectively. We found no evidence of association between either SNP (rs6983267 and rs10808556) and *MYC* (p value > 0.05) or SNP rs4245739 (1q32.1) and *NFASC* (p value = 0.14).

### Functional analysis of candidate SNP regions

**Figure 5:**
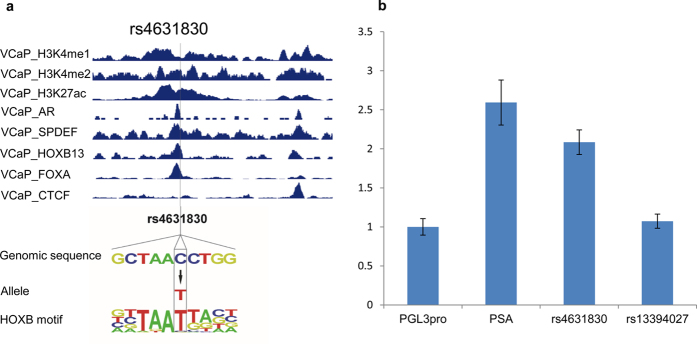
Functional annotation and analysis of SNP rs4631830. (**a**) Epigenetic marks and transcription factor binding at and near rs4631830 in the VCaP cell line. The genomic sequence of rs4631830 and position weighted matrix for HOXB-like DNA binding motif are shown. The rs4631830 base position is indicated by a rectangle. (**b**) Enhancer reporter assay for rs4631830-containing region in LNCaP cell line. Compared to baseline level, both PSA enhancer (positive control) and rs4631830-containing fragment showed increased enhancer activities.

To further refine these SNPs, we examined whether these eQTL SNPs were correlated with the high interaction fragments. We found that rs1446669 (BE5-6 fragment), rs1078004 (BE7-8 fragment) and rs699664 (BE8-9 fragment) at 2p11.2 resided in an interaction peak with the 5′-end of *CAPG*; *C2orf43*-associated SNP rs13394027 was located in the high interaction fragment AE3-4 at 2p24.1. *AGAP7P*-associated SNP rs10993994 and rs4631830 were mapped to high contact fragments E10U and E10D, respectively (Supplementary Table S4). In addition, we also found positive ChIP-seq signals at these SNP regions. For example, in the VCaP cell line, rs1078004 was overlapped with H3K27ac and H3K4me2, and rs144669 site showed a CTCF binding activity. rs4631830 was predicted to disrupt the HOXB-like DNA-binding affinity (Fig. 5a). Consistently, ChIP-seq assays showed strong chromatin binding of HOXB13 together with AR and FOXA1 at this region. In addition, rs4361830-containing region was enriched for active enhancer epigenetic marks including H3K4me1, H3K4me2 and H3K27ac (Fig. 5a). To determine whether SNP-containing fragments have an enhancer activity, we cloned the DNA fragment (517 bp with SNP in the middle) into a pGL3 vector, upstream of the SV40 promoter. The enhancer reporter assay showed 2.1 fold increase when comparing the fragment containing SNP rs4631830 to the control vector PGL3 (Fig. 5b). However, the fragment containing SNP rs13394027 did not show enhancer activity. The enhancer activity of rs4361830-containing region is comparable with the PSA enhancer as a positive control. To further clarify the allelic effect, we further investigated allele-specific enhancer activity of rs4361830-containing fragment by introducing a C → T mutation using site-directed mutagenesis. The results showed that the enhancer activity was higher in risk allele C than common allele A (p value = 8.95E-5) (Supplementary Fig. S5). The reduced enhancer activity in common allele T indicates that the C to T change may disrupt the transcription factors binding.

### Association between target genes and prostate cancer

To examine if these candidate target genes were associated with prostate cancer, we downloaded a TCGA dataset consisting of RNA-seq derived from prostate cancer tissues and normal prostate controls. We compared the normalized RNA-seq values between 498 cancer tissues and 53 benign prostate tissues. We observed significant differences (p values from 0.01 to 6.64E-22) in 15 of 17 selected genes at gene level (Supplementary Table S5). Among these differentially expressed genes, *UBE2Q1, TRIP13, RFX6, TIMM23, MYC, PVT1, CASC8 (LOC727677)* and *AGAP7P* were up-regulated and the remaining *GPRC6A, CAPG, C2orf43, NFASC, KCNN3, PIK3C2B,* and *PPP1R15B* were down-regulated. In particular, the genes *GPRC6A, TRIP13, CAPG, PVT1*, and *C2orf43* demonstrated over 2 fold changes with p value < = 3.56E-10. Further exon level analysis showed that the expression differences in some genes were driven by a portion of entire coding exons (Supplementary Fig. S4). For example, the last four exons of *UBE2Q1* and last five exons of *RFX6* contributed to increased expression levels in prostate cancer tissues. Meanwhile, only one of two exons in *PPP1R15B* contributed to decreased expression. Of interest, the first two and the last two exons of *USP39* showed up-regulation but exons 4–10 demonstrated down-regulation in prostate cancer. This may explain insignificant expression change of the gene at the whole gene level.

### Validation of chromatin interaction at 2p11.2 by 3C-qPCR

**Figure 6:**
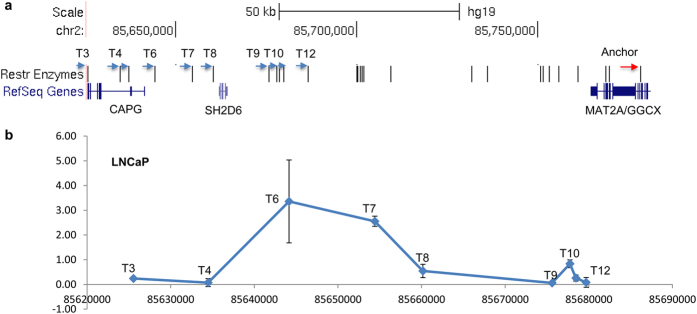
Validation of physical interactions at 2p11.2 locus. (**a**) Physical map of EcoRI sites and 3C-qPCR primers. The anchor primer (red arrow) is near EcoRI site (chr2: 85778503). Nine test primers (blue arrows) are near EcoRI sites from T3 (chr2: 85625540) to T12 (chr2: 85679686) where the *CAPG* promoter and nearby region are located. (**b**) 3C qPCR-based interaction signals between anchor primers and test primers. Error bars represent standard deviation of triplicate qPCR results.

To validate the specific chromatin interactions, we selected one significant locus and performed 3C-qPCR using an anchor primer at bait fragment E9U and test primers at several EcoRI fragments (T3-T12) around *CAPG* gene locus at 2p11.2. The bait fragment E9U was selected because of its higher interaction signal and strong histone modification peaks in addition to transcription factor (FOXA1, HOXB13) binding sites. After normalizing Ct values to an internal randomly ligated control, our 3C-qPCR demonstrated stronger signals at EcoRI fragments containing T6 (chr2: 85644150) and T7 (chr2: 85654414) (Fig. 6), which was consistent with the interaction peak in 3C-seq data.

## Discussion

GWAS have identified thousands of variants associated with hundreds of disease traits. The next critical step in deciphering these associations is to develop high-throughput methods to link cis- regulatory elements to their target genes and determine how these variants alter gene expression. Capture-based 3C-seq, by incorporation of target capture into the 3C-seq technology, allows for the systematic and relatively straightforward identification of the long-range interactions with multiple regulatory regions in one assay^24^,^25^,^26^,^27^,^28^. In this study, we applied 3C-MTS approach to test 10 prostate cancer risk regions for potential cis-interacted target genes in six cell lines. Our study showed that gene regulations through long-range chromatin interactions at the risk loci are common. Although relatively weak, the interaction signals detected by the 3C approach provide additional evidence linking risk SNPs and their candidate target genes.

By applying the capture-based 3C seq, we were able to find the physically interacting genes at risk SNP loci. The candidate target genes included *PPP1R15B/PIK3C2B*, *NFASC* at 1q32.1, *C2orf43* at 2p24.1, *CAPG* at 2p11.2, *RFX6* and *GPRC6A* at 6q22.1, *MYC* and *PVT1* at 8q24.21, *AGAP7P* at 10q11.23. Of those, the most significant and consistent interactions across different cell lines were at *CAPG* locus at 2p11.2*. CAPG* encodes a member of the gelsolin/villin family of actin-regulatory proteins; the encoded protein contributes to the control of actin-based motility in non-muscle cells^40^. *CAPG* is also a candidate tumor suppressor^41^. TCGA data showed 2.67 fold decreased expression in tumor tissues (p = 4.88E−19). (Supplementary Table S5). We found that the physical interaction was not cell type-specific because it was observed in all cell lines tested. However, the interactions at 1q32.1 with *NFASC* were prostate tissue-specific because they were only found in prostate-derived cell lines. *NFASC* encodes an L1 family immunoglobulin cell adhesion molecule with multiple IGcam and fibronectin domains. The protein functions in neurite outgrowth, fasciculation, and organization of the axon initial segment (AIS) and nodes of Ranvier on axons during early development^42^. TCGA data showed 1.65 fold decreased expression in tumor tissues. AGAP7P, a non-coding pseudogene, showed 1.38 fold increased expression in tumor tissues in TCGA data, but the role of this non-coding gene in prostate cancer remains unknown. In fact, 15 of 17 selected genes in these loci show significant expression change in prostate cancer tissues. These results suggest that the genes physically interacting with risk loci are prostate cancer-related.

Although GWAS identified numerous SNPs with an increased risk for PC, there is still a challenge to identify the functional SNPs, largely because the risk SNPs are not necessarily causative. 3C-seq technology was able to define the candidate functional SNPs into one or several restriction fragments. By integrating with eQTL analysis, our study is able to identify candidate SNPs that may regulate target gene expressions. By further integrating with ChIP-seq data, our study is able to refine these candidate SNPs that are overlapped with active histone modifications, and transcription factor binding including FOXA1 and HOXB13, the critical transcription factors during prostate cancer development. Following this workflow, our study has identified several candidate SNPs and their target genes. For example, the DNA fragment containing rs4631830 at 10q11.23 is physically interacted with non-coding gene *AGAP7P*. This interaction appears to be prostate-specific. The SNP rs4631830, located in a chromatin region with multiple transcription factor binding signals, is predicted to disrupt HOXB-like DNA-binding motif and shows enhancer activity. Therefore, integration of 3C-seq, eQTL and epigenomic analyses will facilitate discovery of candidate causal SNPs and their potential target genes.

Our data demonstrated a complex chromatin interaction at the risk loci. Depending on complexity, these long-range interactions may be classified into three models. The first is a one to one model: one predicted regulatory fragment interacts with one specific promoter fragment. An example is 2q11.3 locus where E8-9 fragment shows strong interaction signals at the 5′ end of *CAPG*. The second model is a one to many model: one regulatory fragment interacts with several target genes simultaneously. An example is at the 6q22.1 locus where fragment E16-17 interacts with both *RFX6* and *GPRC6A*. The third is a many to many models; many regulatory fragments interact with multiple genes. Examples include the loci at 1q32.1 for *PIK3C2B* and *NFASC*, and at 8q24 for *MYC* and *PVT1*^26^. These frequent interactions suggest a special chromatin loop structure or several regulatory elements aggregated in multiple fragments to facilitate genes expression. Additionally, we found a LCL-specific TAD at the 8q24 risk region, indicating the importance of the nuclear localization and 3D structure of the genome in cell functions.

Although the capture-based 3C-seq is able to link risk SNPs found in regulatory elements to the target genes, thereby providing a possible regulatory mechanism, there are still some limitations of this technology. The first limitation is weak signal in the detection of significant chromatin interactions. Our data show that interaction signals are often embedded within a large amount of background noises. Sometimes, the signals are hardly distinguishable from nearby background in read count-based heat maps. Because all 3C assays are based on proximity ligation, significant improvement of ligation specificity is technically challenging. Therefore, increasing sequencing depth and improving capture efficiency may partially solve the problem. The second limitation is generally low resolution which makes it difficult for fine mapping of regulatory elements and determination of candidate genes. Our data show that the captured interactions are more likely involved in multiple bait fragments and many continuous fragments at target gene loci. To increase the mapping resolution, one approach is to use more frequent cutter or different restriction enzymes. Another approach is to take advantage of existing epigenomic data from a large variety of resources. Our analysis suggests that interaction peaks are usually accompanied with chromatin modification and/or transcription factor binding sites, indicating that these interaction fragments may contain functional cis-acting elements. Further integration of different approaches into a streamlined workflow will enable translation of GWAS discovery into underlying biological mechanisms.

In summary, this is the first capture-based high throughput 3C study to systematically investigate the long-range chromatin interactions at the multiple prostate cancer risk loci. By integrative analysis of the 3C-seq, eQTL and ChIP-seq data, we were able to define several fragments containing genetic variants that may contribute to expression variation of the candidate genes, hence, increasing the risk of prostate cancer. By examining multiple cell lines, we were able to identify cell line and cell type-specific chromatin interactions. Our study reveals a set of candidate genes and their potential regulatory variants and provides additional evidence showing the role of long-range chromatin interactions in prostate cancer etiology. Further understanding genetic effect and biological mechanism of these chromatin interactions will shed light on the newly discovered regulatory role of the risk loci in prostate cancer etiology and progression.

## Methods

### Target selection and probes design

The target regions for the probe design were based on prostate GWAS risk SNPs reported in http://www.genome.gov/gwastudies^30^,^31^,^43^,^44^,^45^,^46^,^47^,^48^,^49^,^50^. From these publications 10 prostate cancer risk SNPs were chosen (Table S1). In order to choose 10 loci from the list of over 100 GWAS risk loci for this initial experiment, we performed a literature search linking the loci to prostate cancer. We also searched a public dataset for Hi C experiments (http://hic.umassmed.edu/welcome/welcome.php), with the settings of GM06690 cell line and 100 kb resolution observed data for each of the loci, for some evidence of interactions even though the resolution was low. Finally we checked eQTL sources from the U of Chicago (http://eqtl.uchicago.edu/Home.html) for eQTL hits in our loci. We used these limited datasets to identify 10 loci that may have chromatin interactions. For each SNP we determined the best LD block that included each risk SNP and all LD SNPs using the HapMap LD Phased track on the UCSC browser as well as Haploview (https://www.broadinstitute.org/scientific-community/science/programs/medical-and-population-genetics/haploview). The LD SNPs included in each block were defined by choosing the outermost SNPs that were in LD with the risk SNP. We expanded all selected regions to >20 kb by centering on the original LD block. We combined the LD regions when two LD blocks overlapped. Size of the LD regions ranged from 20–476 kb. For each LD region all of the EcoRI sites were mapped using the REBASE (The Restriction Enzyme Database) track on the UCSC browser. At each EcoRI site, 250 bp on each side of the cut site were chosen for the probe design. Agilent Design Service was used to design the probes tiled at 5X. Any probes expanded into or across the EcoRI cut sites were removed.

### 3C-MTS libraries generation

Cell culture, formaldehyde crosslinking, and 3C library preparation were performed as described in Du *et al.*^26^. Targets capture was carried out using the Agilent Bravo liquid handler following the protocol for Agilent’s SureSelect XT. 1 ug of the prepped library was incubated with biotinylated RNA capture baits supplied in the kit for 24 hours at 65 °C. The captured DNA: RNA hybrids were recovered using Dynabeads MyOne Streptavidin T1 from Dynal (Life technologis, Carlsbad, CA, Cat# 65601). The DNA was eluted from the beads and purified using Ampure XP beads from Agencourt. The purified capture products were then amplified using the SureSelect Post-Capture Indexing forward and Index PCR reverse primers for 16 cycles. Libraries were validated and quantified on an Agilent bioanalyzer.

### 3C-MTS library data analysis

Target enriched sequencing libraries were prepared and sequenced on an Illumina Genome Analyzer HiSeq 2000 with 100 bp PE reads. Each library was sequenced in one lane. Bowtie (v.2.0) was used to align each end of the 100 bp pairs to the human hg19 reference sequence; the seed for alignment was 22 bases with no mismatches allowed. A pipeline of PERL scripts was created to extract interactions from the mapped sequences. We excluded the following sequences: both read ends (R1 and R2) mapped to outside of the capture region and duplicate reads aligning to the same genome location. The resulting alignments were assigned to the respective restriction fragments derived from EcoRI digestion sites. Informative sequence pairs were defined as those with at least one end mapped to within 250 bp of the nearest EcoRI sites of target regions. Cis-interactions were defined as one end mapped on the target regions, the other mapped either inside or outside of the probe regions on the same chromosome. To correct for potential differences in probe capture efficiency, read count for each probe region were normalized to median read counts across all probes. The probes which were located in repeat sequences or had low capture efficiency (lower 22 percentile) were removed. The significant interactions at each probe region were determined using fourSig software^29^.

### PCR primers and 3C quantitative PCR (3C-qPCR)

Primers were designed for the detection of interactions between *CAPG* and risk locus 2q11.3. The anchor primer at 2q11.3 was designed at the position (chr2: 85778503) named E9U. Nine test primers were designed on 2q11.3 around nine EcoRI cutting sites from position chr2: 85625540 (T3) to position chr2: 85679686 (T12) which corresponded to the promoter and nearby region of *CAPG* (Fig. 6). Each test primer was paired with the anchor primer. The sequences of the primers are listed in Supplementary Table S6. All PCR reactions were performed using Taqman Universal Master Mix II (Applied Biosystems, Foster City, CA, Cat# 4440038). Each of 10 ul reaction consisted of 1 × Taqman Universal Master Mix II, 1 ul 5 uM anchor primer, 1 ul test primer, 1 ul Taqman probe (2.5 uM) and 100 ng 3C DNA. PCR cycles were as follows: an initial step 2 min at 50 °C, 10 min at 95 °C, 50 cycles of 15 s at 95 °C and 60 s at 60–62 °C. Each PCR reaction was performed in triplicate, and the data presented were the average of at least two independent experiment results for all PCR reactions. The contact frequency of each interaction pair was normalized using a 3C-control library prepared from pooled PCR products that contained 16 EcoRI-digested and T4 ligase-ligated fragments covering target EcoRI cutting sites and primer binding sites. Adjacent fragment ligation frequency was used to normalize the different loading, fixation and ligation efficiencies between different cell lines (63, 64).

### Epigenomic analysis and eQTL analysis

To evaluate functional relevance of the chromatin interactions, we aligned our interaction peaks with the genomic locations of GENCODE genes (v19; http://www.gencodegenes.org), and histone modifications including active enhancers (H3K27ac, H3K4me1), active promoters (H3K4me3) and (H3K4me2), which usually overlapped with transcription factor binding regions^51^,^52^,^53^,^54^. For the most significant interactions, we examined the binding of genome organizer proteins CTCF, RAD21 and prostate specific transcription factors: AR, FOXA1, HOXB13 in prostate specific cell lines LNCaP, VCaP and NCIH660. eQTL analysis was performed using RNA-seq data and SNP genotyping data (Illumina HumanOmni2.5–8 Beadchip) derived from 476 normal prostate tissues in our laboratory. Linear regression was used to correlate genotypes of selected SNPs and expression level (RPKM) of candidate genes.

### Enhancer reporter assay

SNPs rs4631830 and rs13394027 were selected for enhancer reporter assay. The SNP-centered DNA fragment (~500 bp) was amplified from human genomic DNA and cloned into upstream of the SV40 promoter in the pGL3 promoter vector (Promega, Madison, WI). For allele-specific enhancer activity, site-directed mutagenesis (Stratagene, La Jolla, CA) was used to create allele-specific construct. The oligo sequences used in this mutagenesis were listed in Supplementary Table S6. In addition, the PSA (prostate-specific antigen) enhancer-containing fragment (~600 bp) was used as a positive control. Plasmid DNAs were prepared and purified using QIAprep Miniprep kit (Qiagen,Valencia,CA). For plasmid transfection on white 96-well tissue culture plates, 100 ul suspension (3 × 10^5^ cells/ml) of LNCaP cells per well were applied to reverse transfection with luciferase reporter plasmids together with pGL75 using X-tremeGENE HP DNA Transfection Reagent (Roche Applied Science, Penzberg, Upper Bavaria, Germany) following the manufacturer’s instructions. 48 hours later, cells were analyzed for luciferase activities using Dual-Glo® Luciferase Assay System (Promega). All data came from five replicate wells.

## Additional Information

**How to cite this article**: Du, M. *et al.* Chromatin interactions and candidate genes at ten prostate cancer risk loci. *Sci. Rep.* **6**, 23202; doi: 10.1038/srep23202 (2016).

## Supplementary Material

Supplementary Information

## Figures and Tables

**Table 1:** Statistics for seven 3C-MTS libraries.

Cell Lines	LnCaP-1	LnCaP-2	DU-145	BPH-1	RWPE-1	LCL-29092	LCL-32370	Average
raw_reads_pairs	183,910,322	198,631,666	194,315,748	210,756,996	194,265,309	202,529,498	196,554,517	197,167,026
Unmapped sequence pairs	40,690,682	37,168,884	35,978,070	35,107,246	39,147,398	35,206,879	32,411,075	34,924,646
Mapped sequence pairs	143,219,640	161,462,782	158,337,678	175,649,750	155,117,911	167,322,619	164,143,442	162,242,380
Outside target regions	119,949,803	135,712,575	102,522,249	118,421,990	115,548,493	128,135,295	128,015,247	124,067,690
On target regions	23,269,837	25,750,207	55,815,429	57,227,760	39,569,418	39,187,324	36,128,195	38,174,690
Cis_interactions	20,754,813	23,340,515	50,216,052	52,175,436	34,712,028	35,401,140	32,694,543	34,707,547
Trans_interactions	2,515,024	2,409,692	5,599,377	5,052,324	4,857,390	3,786,184	3,433,652	3,467,143
% on target pairs	16.2%	15.9%	35.3%	32.6%	25.5%	23.4%	22.0%	23.4%
% Cis	89.19%	90.64%	89.97%	91.17%	87.72%	90.34%	90.50%	91.2%
% Trans	12.12%	10.32%	11.15%	9.68%	13.99%	10.70%	10.50%	9.8%

**Table 2:** The most common and significant chromatin interactions.

Chr. Loci	Bait regions	Target Genes	Positions in gene	Cell Lines
1q21.3	AE5-7; AE11-12	UBE2Q1	5′-end	LNCaP, DU-145, BPH-1, RWPE-1, LCL
1q21.3	AE5-7; AE11-12	KCNN3	3′-end; Introns	LNCaP, DU-145, BPH-1, RWPE-1, LCL
1q32.1	BE5-6; BE9-10	PPP1R15B	5′-end	LNCaP, DU-145, BPH-1, RWPE-1, LCL
1q32.1	BE5-6; BE9-10	PIK3C2B	3′-end; Introns	LNCaP, DU-145, BPH-1, RWPE-1, LCL
1q32.1	BE15-18; BE23-25	NFASC	5′-end	LNCaP, DU-145, BPH-1, RWPE-1
2p24.1	AE3-4; AE6-7	HS1BP3	5′-end	LNCaP, DU-145, BPH-1, RWPE-1, LCL
2p24.1	AE3-4; AE6-7	C2orf43	5′-end; Intron 4	LNCaP, DU-145, BPH-1, RWPE-1
2p11.2	BE6-9	CAPG	5′-end	LNCaP, DU-145, BPH-1, RWPE-1, LCL
2p11.2	BE14-15	USP39	5′-end	LNCaP, DU-145, BPH-1, RWPE-1, LCL
5p15.33	AE2-3	TRIP13	5′-end	LNCaP, DU-145, BPH-1, RWPE-1, LCL
5p15.33	AE2-3	LOC100506688	3′-end	LNCaP, DU-145, BPH-1, RWPE-1
6q22.1	E16-17	RFX6	5′-end	LNCaP, DU-145, BPH-1, RWPE-1
6q22.1	E16-17	GPRC6A	5′-end	LNCaP, BPH-1, RWPE-1
8q24.21	E71-75; E92-93; E128-129	MYC	5′-end	LNCaP, DU-145, BPH-1, RWPE-1
8q24.21	E39-42; E71-75	MYC	5′-end	LCL
8q24.21	E92-93; E128-129	PVT1	5′-end	LNCaP
8q24.21	E6-7; E42-43; E92-93; E128-129	PCAT1	5′-end	LNCaP, DU-145
8q24.21	E26-27; E39-42; E71-75	CASC8	5′-end	LCL
10q11.23	E6-7; E9-10	AGAP7	5′-end	LNCaP, DU-145, BPH-1, RWPE-1
10q11.23	E6-7; E9-10	ANTXRLP1	5′-end	LNCaP, DU-145, BPH-1, RWPE-1
11q13.2	E4-5; E12-13	LOC338694	5′-end	LNCaP, DU-145, BPH-1, RWPE-1, LCL

Chr: chromosome; E: EcoRI digestion site; U: upstream; D: downstream; A: the first region on the chr; B: the second region on the same chr.
