# Supplementary material for: Chromatin interactions and candidate genes at ten prostate cancer risk loci
Source: Sci Rep. 2016 Mar 16;6:23202. doi: 10.1038/srep23202 (PMC4793270; doi:10.1038/srep23202)
Supplement: Supplementary Information [file srep23202-s1.pdf]

Chromatin interactions and candidate genes at ten prostate cancer risk loci

Meijun Du[1], Lori Tillmans[2], Jianzhong Gao[3], Ping Gao[5], Tiezheng Yuan[1], Rachel L Dittmar[1], Wei Song[5], Yuehong Yang[5], Natasha Sahr[4], Tao Wang[4], Gong-Hong Wei[5], Stephen N. Thibodeau[2], Liang Wang[1,] *

Supplementary figures

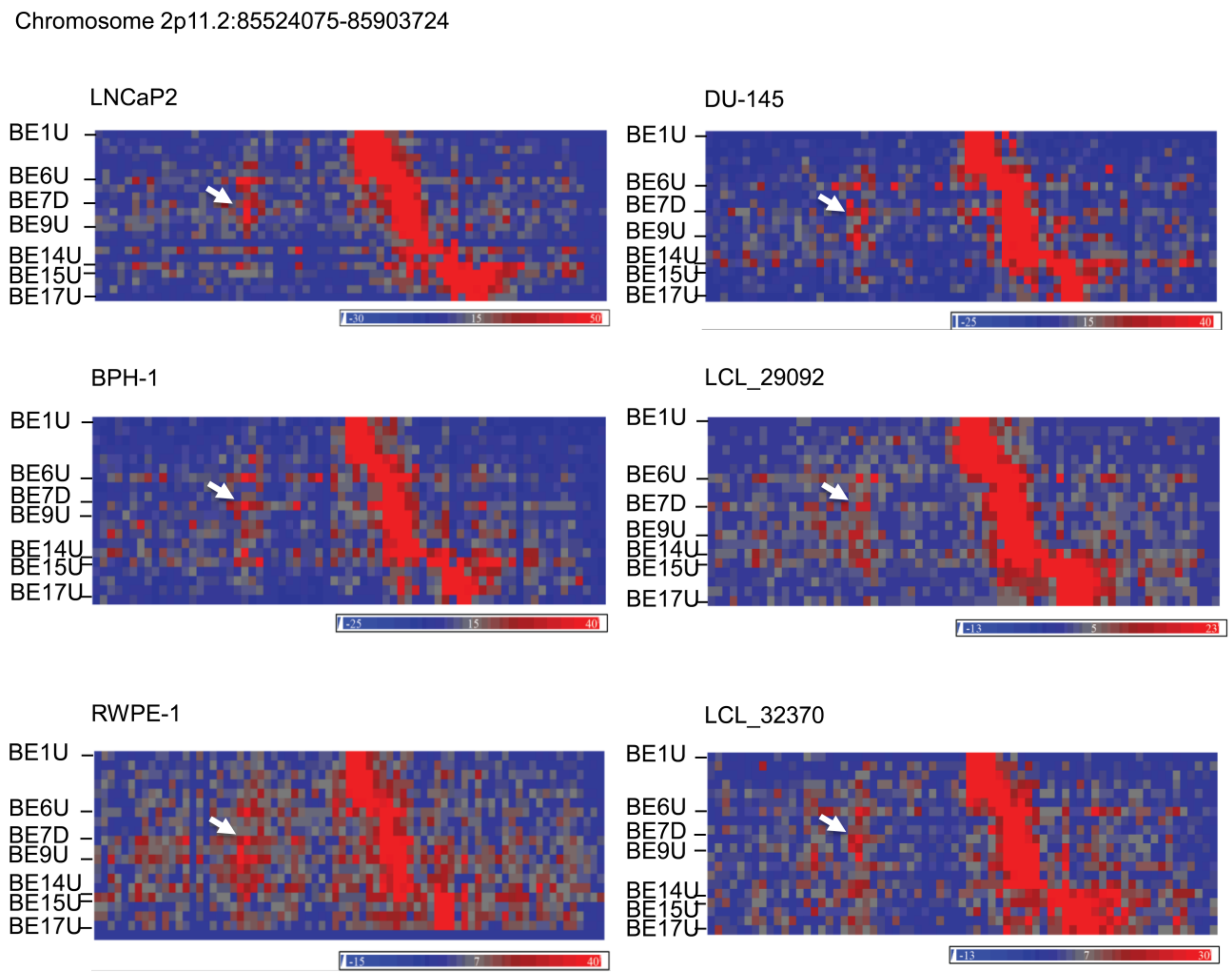

Supplementary Fig. S1. Physical interaction heatmaps at 2p11.2 locus in six cell lines. Y-axis lists 17 EcoRI sites where 22 bait fragments are shown from E1 (chr2:85750614) to E17

(chr2:85811383). X-axis is EcoRI-defined fragments from chr2: 85524075 to 85903724 (66 EcoRI cutting sites). White arrows indicate interaction hot spots.

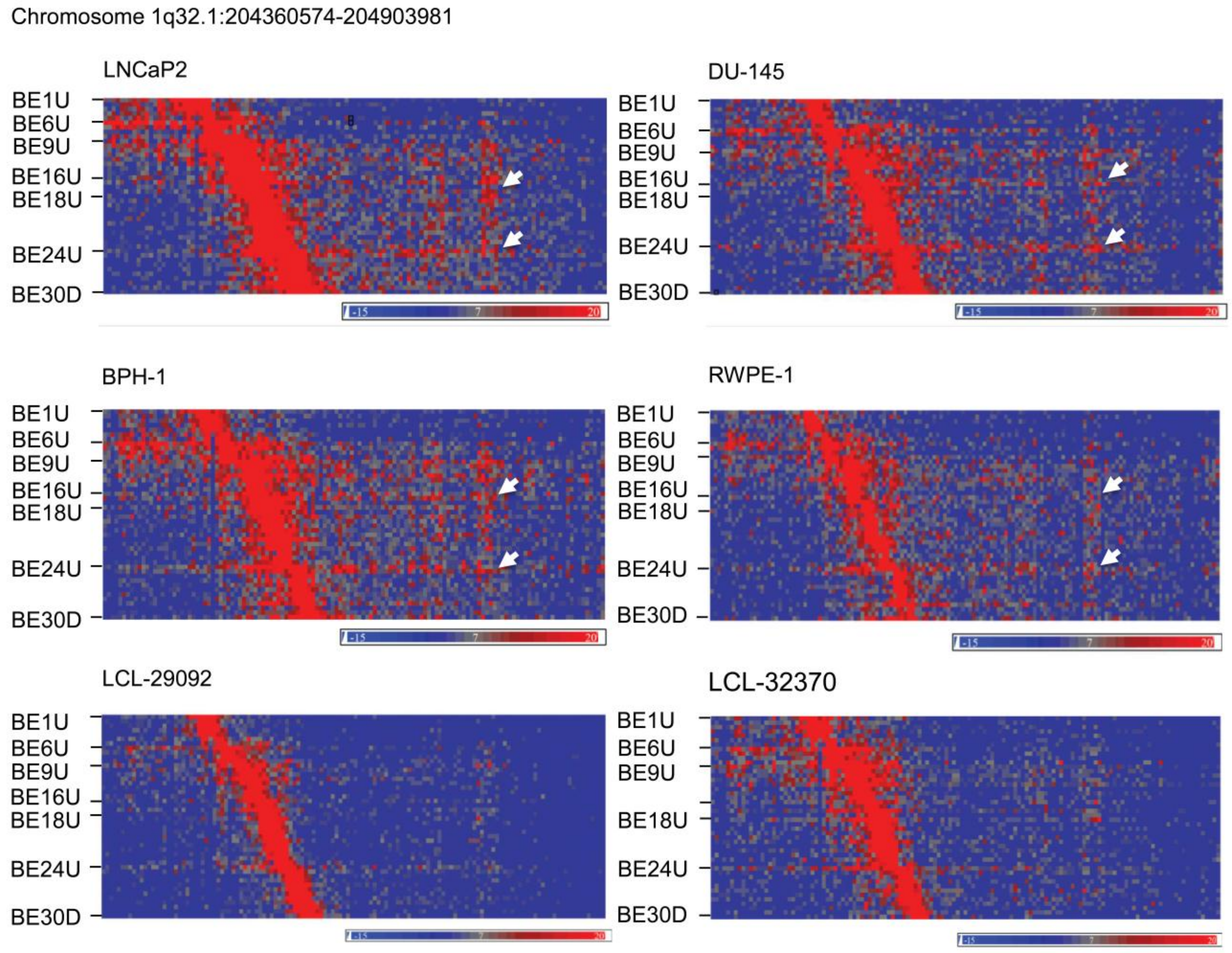

Supplementary Fig. S2. Physical interaction heatmaps at 1q32.1 locus in six cell lines. Y-axis lists 30 EcoRI sites where 46 bait fragments are shown from chr1:204457557 to 204571652. X-axis is EcoRI-defined fragments from chr1: 204360574 to 204903981 (135EcoRI cutting sites). White arrows indicate interaction hot spots.

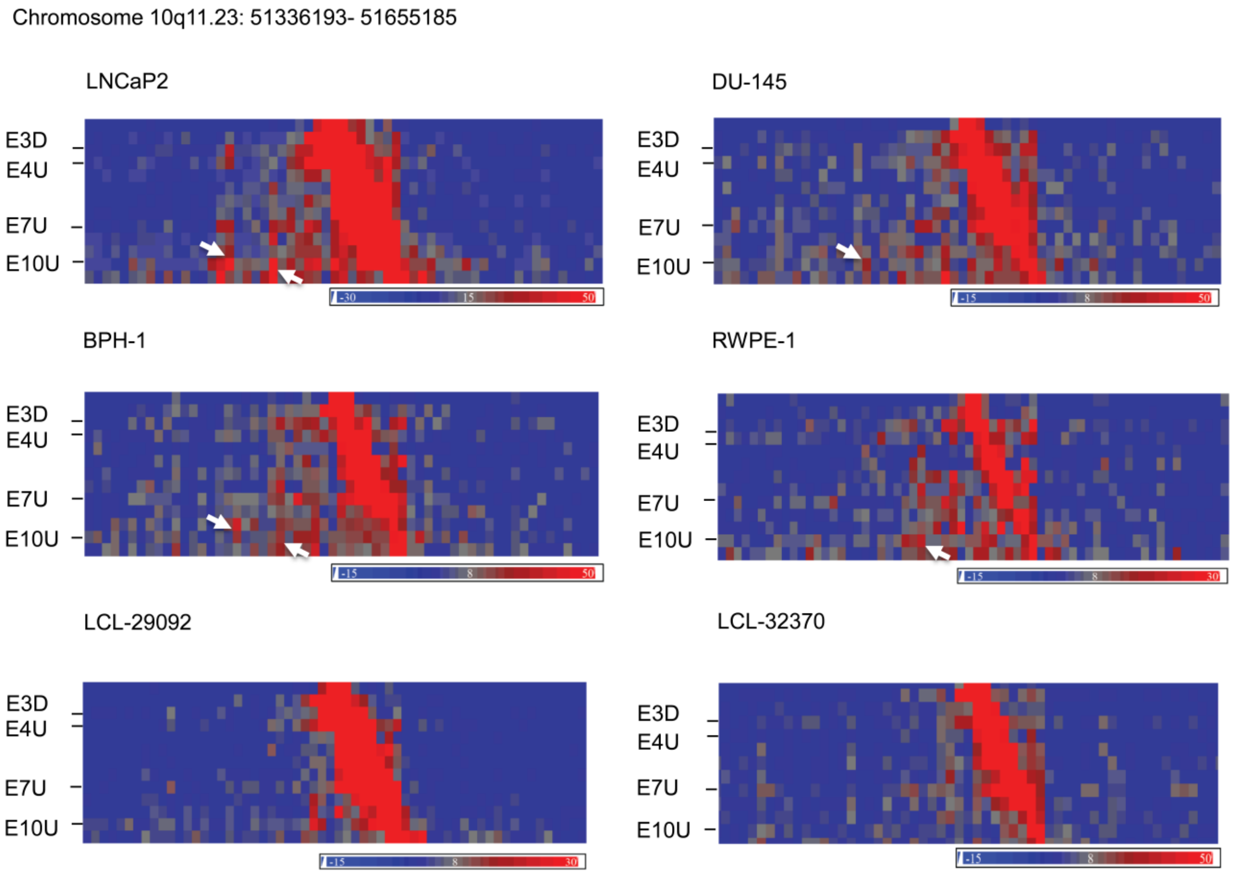

Supplementary Fig. S3. Physical interaction heatmaps at 10q11.23 locus in six cell lines. Y-axis lists 10 EcoRI sites where 13 bait fragments are shown from chr10: 51514825 to 51547645. X-axis is EcoRI-defined fragments from chr10: 51336193 to 51655185 (60EcoRI cutting sites). White arrows indicate interaction hot spots.

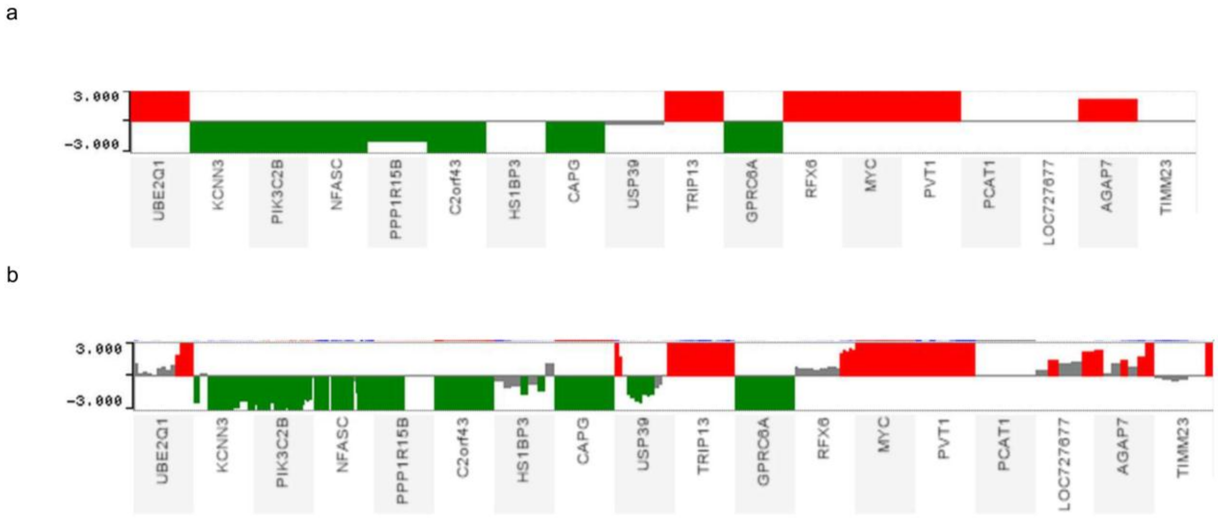

Supplementary Fig. S4. Target gene expression changes between prostate cancer and normal tissues. TCGA Cancer Browser was used to generate the expression changes. Green or red bars represent statistically significant down- or up-regulation in cancer tissues, respectively. Grey bars represent no significant changes. A. expression changes are shown at whole gene level. B. expression changes are shown in exon level.

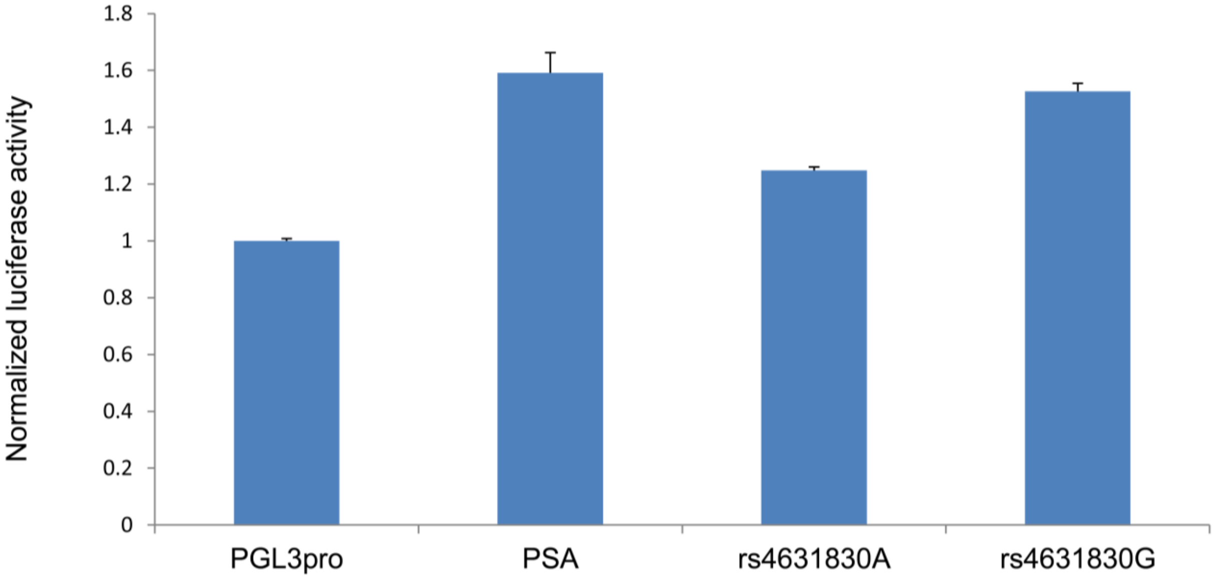

Supplementary Fig. S5. Allele-specific enhancer reporter assay for rs4631830-containing Fragment in LNCaP cell line. Site-directed mutagenesis was performed to make a point mutation from C to T in the rs4631830 containing fragment. The luciferase-based results showed that both alleles C and T increased enhancer activity when compared to baseline vector control PGL3pro. Importantly, the risk allele C demonstrated stronger enhancer activity than common allele T (p=8.95E-5).

Supplementary tables

Supplementary Table S1 Targeted risk loci and genomic coordinates

Risk Loci	Representative SNPs	LD blocks (Coordinates)	Size(kb)	EcoRI sites
1q21.3	rs1218582	chr1:154831376-154918376	87	14
1q32.1	rs4245739	chr1:204456377-204576377	120	30
2p24.1	rs13385191	chr2:20877519-20897519	20	7
2p11.2	rs2028898/rs10187424	chr2:85738489-85811489	73	17
5p15.33	rs2242652	chr5:1272999-1303000	20	3
5p12	rs2121875	chr5:44266243-44393243	127	36
6q22.1	rs339331	chr6:117209307-117281307	72	28
8q24.21	rs12543663	chr8:128069818-128551818	482	135
	rs10086908			
	rs16901979			
	rs10808556			
	rs6983267			
	rs1447295			
10q11.23	rs10993994/rs4631830	chr10:51502994-51549994	47	10
11q13.2	rs7931342	chr11:68974424-69038424	64	20

Supplementary Table S2. Probe sites for ten risk regions

Probe Names	Chr.	EcoRI sites	Probe regions
chr1_AE1U	chr1	154832652	154832402-154832652
chr1_AE1D	chr1	154832652	154832652-154832902
chr1_AE2U	chr1	154835814	154835564-154835814
chr1_AE2D	chr1	154835814	154835814-154836064
chr1_AE3U	chr1	154841258	154841008-154841258
chr1_AE3D	chr1	154841258	154841258-154841508
chr1_AE4U	chr1	154843978	154843728-154843978
chr1_AE4D	chr1	154843978	154843978-154844228
chr1_AE5U	chr1	154844039	154843789-154844039
chr1_AE5D	chr1	154844039	154844039-154844289
chr1_AE6U	chr1	154848585	154848335-154848585
chr1_AE6D	chr1	154848585	154848585-154848835
chr1_AE7U	chr1	154859292	154859042-154859292
chr1_AE7D	chr1	154859292	154859292-154859542
chr1_AE8U	chr1	154881213	154880963-154881213
chr1_AE8D	chr1	154881213	154881213-154881463
chr1_AE9U	chr1	154881945	154881695-154881945
chr1_AE9D	chr1	154881945	154881945-154882195
chr1_AE10U	chr1	154883687	154883437-154883687
chr1_AE10D	chr1	154883687	154883687-154883937
chr1_AE11U	chr1	154888031	154887781-154888031
chr1_AE11D	chr1	154888031	154888031-154888281
chr1_AE12U	chr1	154904153	154903903-154904153
chr1_AE12D	chr1	154904153	154904153-154904403
chr1_AE13U	chr1	154911415	154911165-154911415
chr1_AE13D	chr1	154911415	154911415-154911665
chr1_AE14U	chr1	154916057	154915807-154916057
chr1_AE14D	chr1	154916057	154916057-154916307
chr1_BE1U	chr1	204457557	204457307-204457557
chr1_BE1D	chr1	204457557	204457557-204457807
chr1_BE2U	chr1	204459511	204459261-204459511
chr1_BE2D	chr1	204459511	204459511-204459761
chr1_BE3U	chr1	204460225	204459975-204460225
chr1_BE3D	chr1	204460225	204460225-204460475
chr1_BE4U	chr1	204460530	204460280-204460530
chr1_BE4D	chr1	204460530	204460530-204460780
chr1_BE5U	chr1	204468627	204468377-204468627

chr1_BE5D	chr1	204468627	204468627-204468877
chr1_BE6U	chr1	204475811	204475561-204475811
chr1_BE6D	chr1	204475811	204475811-204476061
chr1_BE7U	chr1	204476953	204476703-204476953
chr1_BE7D	chr1	204476953	204476953-204477203
chr1_BE8U	chr1	204479635	204479385-204479635
chr1_BE8D	chr1	204479635	204479635-204479885
chr1_BE9U	chr1	204482247	204481997-204482247
chr1_BE9D	chr1	204482247	204482247-204482497
chr1_BE10U	chr1	204489040	204488790-204489040
chr1_BE10D	chr1	204489040	204489040-204489290
chr1_BE11U	chr1	204496085	204495835-204496085
chr1_BE11D	chr1	204496085	204496085-204496335
chr1_BE12U	chr1	204507727	204507477-204507727
chr1_BE12D	chr1	204507727	204507727-204507977
chr1_BE13U	chr1	204512162	204511912-204512162
chr1_BE13D	chr1	204512162	204512162-204512412
chr1_BE14U	chr1	204517321	204517071-204517321
chr1_BE14D	chr1	204517321	204517321-204517571
chr1_BE15U	chr1	204518514	204518264-204518514
chr1_BE15D	chr1	204518514	204518514-204518764
chr1_BE16U	chr1	204532039	204531789-204532039
chr1_BE16D	chr1	204532039	204532039-204532289
chr1_BE17U	chr1	204534875	204534625-204534875
chr1_BE17D	chr1	204534875	204534875-204535125
chr1_BE18U	chr1	204536408	204536158-204536408
chr1_BE18D	chr1	204536408	204536408-204536658
chr1_BE19U	chr1	204537965	204537715-204537965
chr1_BE19D	chr1	204537965	204537965-204538215
chr1_BE20U	chr1	204540984	204540734-204540984
chr1_BE20D	chr1	204540984	204540984-204541234
chr1_BE21U	chr1	204541173	204540923-204541173
chr1_BE21D	chr1	204541173	204541173-204541423
chr1_BE22U	chr1	204542849	204542599-204542849
chr1_BE22D	chr1	204542849	204542849-204543099
chr1_BE23U	chr1	204543667	204543417-204543667
chr1_BE23D	chr1	204543667	204543667-204543917
chr1_BE24U	chr1	204550984	204550734-204550984
chr1_BE24D	chr1	204550984	204550984-204551234
chr1_BE25U	chr1	204561889	204561639-204561889
chr1_BE25D	chr1	204561889	204561889-204562139
chr1_BE26U	chr1	204562983	204562733-204562983
chr1_BE26D	chr1	204562983	204562983-204563233

chr1_BE27U	chr1	204564942	204564692-204564942
chr1_BE27D	chr1	204564942	204564942-204565192
chr1_BE28U	chr1	204565862	204565612-204565862
chr1_BE28D	chr1	204565862	204565862-204566112
chr1_BE29U	chr1	204570894	204570644-204570894
chr1_BE29D	chr1	204570894	204570894-204571144
chr1_BE30U	chr1	204571652	204571402-204571652
chr1_BE30D	chr1	204571652	204571652-204571902
chr2_AE1U	chr2	20877964	20877714-20877964
chr2_AE1D	chr2	20877964	20877964-20878214
chr2_AE2U	chr2	20878354	20878104-20878354
chr2_AE2D	chr2	20878354	20878354-20878604
chr2_AE3U	chr2	20879972	20879722-20879972
chr2_AE3D	chr2	20879972	20879972-20880222
chr2_AE4U	chr2	20885578	20885328-20885578
chr2_AE4D	chr2	20885578	20885578-20885828
chr2_AE5U	chr2	20888089	20887839-20888089
chr2_AE5D	chr2	20888089	20888089-20888339
chr2_AE6U	chr2	20889115	20888865-20889115
chr2_AE6D	chr2	20889115	20889115-20889365
chr2_AE7U	chr2	20896362	20896112-20896362
chr2_AE7D	chr2	20896362	20896362-20896612
chr2_BE1U	chr2	85750614	85750364-85750614
chr2_BE1D	chr2	85750614	85750614-85750864
chr2_BE2U	chr2	85751367	85751117-85751367
chr2_BE2D	chr2	85751367	85751367-85751617
chr2_BE3U	chr2	85753043	85752793-85753043
chr2_BE3D	chr2	85753043	85753043-85753293
chr2_BE4U	chr2	85755774	85755524-85755774
chr2_BE4D	chr2	85755774	85755774-85756024
chr2_BE5U	chr2	85761062	85760812-85761062
chr2_BE5D	chr2	85761062	85761062-85761312
chr2_BE6U	chr2	85768683	85768433-85768683
chr2_BE6D	chr2	85768683	85768683-85768933
chr2_BE7U	chr2	85769616	85769366-85769616
chr2_BE7D	chr2	85769616	85769616-85769866
chr2_BE8U	chr2	85778503	85778253-85778503
chr2_BE8D	chr2	85778503	85778503-85778753
chr2_BE9U	chr2	85781283	85781033-85781283
chr2_BE9D	chr2	85781283	85781283-85781533
chr2_BE10U	chr2	85784919	85784669-85784919
chr2_BE10D	chr2	85784919	85784919-85785169
chr2_BE11U	chr2	85792330	85792080-85792330

chr2_BE11D	chr2	85792330	85792330-85792580
chr2_BE12U	chr2	85792803	85792553-85792803
chr2_BE12D	chr2	85792803	85792803-85793053
chr2_BE13U	chr2	85798635	85798385-85798635
chr2_BE13D	chr2	85798635	85798635-85798885
chr2_BE14U	chr2	85804733	85804483-85804733
chr2_BE14D	chr2	85804733	85804733-85804983
chr2_BE15U	chr2	85805038	85804788-85805038
chr2_BE15D	chr2	85805038	85805038-85805288
chr2_BE16U	chr2	85807712	85807462-85807712
chr2_BE16D	chr2	85807712	85807712-85807962
chr2_BE17U	chr2	85811383	85811133-85811383
chr2_BE17D	chr2	85811383	85811383-85811633
chr5_AE1U	chr5	1275548	1275298-1275548
chr5_AE1D	chr5	1275548	1275548-1275798
chr5_AE2U	chr5	1277400	1277150-1277400
chr5_AE2D	chr5	1277400	1277400-1277650
chr5_AE3U	chr5	1299098	1298848-1299098
chr5_AE3D	chr5	1299098	1299098-1299348
chr5_BE1U	chr5	44266735	44266485-44266735
chr5_BE1D	chr5	44266735	44266735-44266985
chr5_BE2U	chr5	44272411	44272161-44272411
chr5_BE2D	chr5	44272411	44272411-44272661
chr5_BE3U	chr5	44273634	44273384-44273634
chr5_BE3D	chr5	44273634	44273634-44273884
chr5_BE4U	chr5	44277090	44276840-44277090
chr5_BE4D	chr5	44277090	44277090-44277340
chr5_BE5U	chr5	44278138	44277888-44278138
chr5_BE5D	chr5	44278138	44278138-44278388
chr5_BE6U	chr5	44278975	44278725-44278975
chr5_BE6D	chr5	44278975	44278975-44279225
chr5_BE7U	chr5	44279486	44279236-44279486
chr5_BE7D	chr5	44279486	44279486-44279736
chr5_BE8U	chr5	44279667	44279417-44279667
chr5_BE8D	chr5	44279667	44279667-44279917
chr5_BE9U	chr5	44283369	44283119-44283369
chr5_BE9D	chr5	44283369	44283369-44283619
chr5_BE10U	chr5	44298492	44298242-44298492
chr5_BE10D	chr5	44298492	44298492-44298742
chr5_BE11U	chr5	44302255	44302005-44302255
chr5_BE11D	chr5	44302255	44302255-44302505
chr5_BE12U	chr5	44305951	44305701-44305951
chr5_BE12D	chr5	44305951	44305951-44306201

chr5_BE13U	chr5	44310857	44310607-44310857
chr5_BE13D	chr5	44310857	44310857-44311107
chr5_BE14U	chr5	44312768	44312518-44312768
chr5_BE14D	chr5	44312768	44312768-44313018
chr5_BE15U	chr5	44315115	44314865-44315115
chr5_BE15D	chr5	44315115	44315115-44315365
chr5_BE16U	chr5	44316612	44316362-44316612
chr5_BE16D	chr5	44316612	44316612-44316862
chr5_BE17U	chr5	44318057	44317807-44318057
chr5_BE17D	chr5	44318057	44318057-44318307
chr5_BE18U	chr5	44335571	44335321-44335571
chr5_BE18D	chr5	44335571	44335571-44335821
chr5_BE19U	chr5	44337381	44337131-44337381
chr5_BE19D	chr5	44337381	44337381-44337631
chr5_BE20U	chr5	44337437	44337187-44337437
chr5_BE20D	chr5	44337437	44337437-44337687
chr5_BE21U	chr5	44342635	44342385-44342635
chr5_BE21D	chr5	44342635	44342635-44342885
chr5_BE22U	chr5	44348735	44348485-44348735
chr5_BE22D	chr5	44348735	44348735-44348985
chr5_BE23U	chr5	44348925	44348675-44348925
chr5_BE23D	chr5	44348925	44348925-44349175
chr5_BE24U	chr5	44357554	44357304-44357554
chr5_BE24D	chr5	44357554	44357554-44357804
chr5_BE25U	chr5	44360606	44360356-44360606
chr5_BE25D	chr5	44360606	44360606-44360856
chr5_BE26U	chr5	44361133	44360883-44361133
chr5_BE26D	chr5	44361133	44361133-44361383
chr5_BE27U	chr5	44363298	44363048-44363298
chr5_BE27D	chr5	44363298	44363298-44363548
chr5_BE28U	chr5	44364080	44363830-44364080
chr5_BE28D	chr5	44364080	44364080-44364330
chr5_BE29U	chr5	44364740	44364490-44364740
chr5_BE29D	chr5	44364740	44364740-44364990
chr5_BE30U	chr5	44367234	44366984-44367234
chr5_BE30D	chr5	44367234	44367234-44367484
chr5_BE31U	chr5	44372719	44372469-44372719
chr5_BE31D	chr5	44372719	44372719-44372969
chr5_BE32U	chr5	44381340	44381090-44381340
chr5_BE32D	chr5	44381340	44381340-44381590
chr5_BE33U	chr5	44383618	44383368-44383618
chr5_BE33D	chr5	44383618	44383618-44383868
chr5_BE34U	chr5	44386704	44386454-44386704

chr5_BE34D	chr5	44386704	44386704-44386954
chr5_BE35U	chr5	44387112	44386862-44387112
chr5_BE35D	chr5	44387112	44387112-44387362
chr5_BE36U	chr5	44392227	44391977-44392227
chr5_BE36D	chr5	44392227	44392227-44392477
chr6_E1U	chr6	117209357	117209107-117209357
chr6_E1D	chr6	117209357	117209357-117209607
chr6_E2U	chr6	117213570	117213320-117213570
chr6_E2D	chr6	117213570	117213570-117213820
chr6_E3U	chr6	117216483	117216233-117216483
chr6_E3D	chr6	117216483	117216483-117216733
chr6_E4U	chr6	117218713	117218463-117218713
chr6_E4D	chr6	117218713	117218713-117218963
chr6_E5U	chr6	117222643	117222393-117222643
chr6_E5D	chr6	117222643	117222643-117222893
chr6_E6U	chr6	117225087	117224837-117225087
chr6_E6D	chr6	117225087	117225087-117225337
chr6_E7U	chr6	117228624	117228374-117228624
chr6_E7D	chr6	117228624	117228624-117228874
chr6_E8U	chr6	117228777	117228527-117228777
chr6_E8D	chr6	117228777	117228777-117229027
chr6_E9U	chr6	117232152	117231902-117232152
chr6_E9D	chr6	117232152	117232152-117232402
chr6_E10U	chr6	117239756	117239506-117239756
chr6_E10D	chr6	117239756	117239756-117240006
chr6_E11U	chr6	117243706	117243456-117243706
chr6_E11D	chr6	117243706	117243706-117243956
chr6_E12U	chr6	117244994	117244744-117244994
chr6_E12D	chr6	117244994	117244994-117245244
chr6_E13U	chr6	117245947	117245697-117245947
chr6_E13D	chr6	117245947	117245947-117246197
chr6_E14U	chr6	117246911	117246661-117246911
chr6_E14D	chr6	117246911	117246911-117247161
chr6_E15U	chr6	117255379	117255129-117255379
chr6_E15D	chr6	117255379	117255379-117255629
chr6_E16U	chr6	117260794	117260544-117260794
chr6_E16D	chr6	117260794	117260794-117261044
chr6_E17U	chr6	117266000	117265750-117266000
chr6_E17D	chr6	117266000	117266000-117266250
chr6_E18U	chr6	117270313	117270063-117270313
chr6_E18D	chr6	117270313	117270313-117270563
chr6_E19U	chr6	117270909	117270659-117270909
chr6_E19D	chr6	117270909	117270909-117271159

chr6_E20U	chr6	117271646	117271396-117271646
chr6_E20D	chr6	117271646	117271646-117271896
chr6_E21U	chr6	117272768	117272518-117272768
chr6_E21D	chr6	117272768	117272768-117273018
chr6_E22U	chr6	117272843	117272593-117272843
chr6_E22D	chr6	117272843	117272843-117273093
chr6_E23U	chr6	117273812	117273562-117273812
chr6_E23D	chr6	117273812	117273812-117274062
chr6_E24U	chr6	117274334	117274084-117274334
chr6_E24D	chr6	117274334	117274334-117274584
chr6_E25U	chr6	117275976	117275726-117275976
chr6_E25D	chr6	117275976	117275976-117276226
chr6_E26U	chr6	117277664	117277414-117277664
chr6_E26D	chr6	117277664	117277664-117277914
chr6_E27U	chr6	117277861	117277611-117277861
chr6_E27D	chr6	117277861	117277861-117278111
chr6_E28U	chr6	117278416	117278166-117278416
chr6_E28D	chr6	117278416	117278416-117278666
chr8_E1U	chr8	128077371	128077121-128077371
chr8_E1D	chr8	128077371	128077371-128077621
chr8_E2U	chr8	128080463	128080213-128080463
chr8_E2D	chr8	128080463	128080463-128080713
chr8_E3U	chr8	128087248	128086998-128087248
chr8_E3D	chr8	128087248	128087248-128087498
chr8_E4U	chr8	128091304	128091054-128091304
chr8_E4D	chr8	128091304	128091304-128091554
chr8_E5U	chr8	128092257	128092007-128092257
chr8_E5D	chr8	128092257	128092257-128092507
chr8_E6U	chr8	128095364	128095114-128095364
chr8_E6D	chr8	128095364	128095364-128095614
chr8_E7U	chr8	128114677	128114427-128114677
chr8_E7D	chr8	128114677	128114677-128114927
chr8_E8U	chr8	128115929	128115679-128115929
chr8_E8D	chr8	128115929	128115929-128116179
chr8_E9U	chr8	128131463	128131213-128131463
chr8_E9D	chr8	128131463	128131463-128131713
chr8_E10U	chr8	128132266	128132016-128132266
chr8_E10D	chr8	128132266	128132266-128132516
chr8_E11U	chr8	128138345	128138095-128138345
chr8_E11D	chr8	128138345	128138345-128138595
chr8_E12U	chr8	128143008	128142758-128143008
chr8_E12D	chr8	128143008	128143008-128143258
chr8_E13U	chr8	128149827	128149577-128149827

chr8_E13D	chr8	128149827	128149827-128150077
chr8_E14U	chr8	128153906	128153656-128153906
chr8_E14D	chr8	128153906	128153906-128154156
chr8_E15U	chr8	128156624	128156374-128156624
chr8_E15D	chr8	128156624	128156624-128156874
chr8_E16U	chr8	128163089	128162839-128163089
chr8_E16D	chr8	128163089	128163089-128163339
chr8_E17U	chr8	128166532	128166282-128166532
chr8_E17D	chr8	128166532	128166532-128166782
chr8_E18U	chr8	128168177	128167927-128168177
chr8_E18D	chr8	128168177	128168177-128168427
chr8_E19U	chr8	128170741	128170491-128170741
chr8_E19D	chr8	128170741	128170741-128170991
chr8_E20U	chr8	128171157	128170907-128171157
chr8_E20D	chr8	128171157	128171157-128171407
chr8_E21U	chr8	128173300	128173050-128173300
chr8_E21D	chr8	128173300	128173300-128173550
chr8_E22U	chr8	128176039	128175789-128176039
chr8_E22D	chr8	128176039	128176039-128176289
chr8_E23U	chr8	128177277	128177027-128177277
chr8_E23D	chr8	128177277	128177277-128177527
chr8_E24U	chr8	128181068	128180818-128181068
chr8_E24D	chr8	128181068	128181068-128181318
chr8_E25U	chr8	128189750	128189500-128189750
chr8_E25D	chr8	128189750	128189750-128190000
chr8_E26U	chr8	128192003	128191753-128192003
chr8_E26D	chr8	128192003	128192003-128192253
chr8_E27U	chr8	128193531	128193281-128193531
chr8_E27D	chr8	128193531	128193531-128193781
chr8_E28U	chr8	128194972	128194722-128194972
chr8_E28D	chr8	128194972	128194972-128195222
chr8_E29U	chr8	128199400	128199150-128199400
chr8_E29D	chr8	128199400	128199400-128199650
chr8_E30U	chr8	128208448	128208198-128208448
chr8_E30D	chr8	128208448	128208448-128208698
chr8_E31U	chr8	128208831	128208581-128208831
chr8_E31D	chr8	128208831	128208831-128209081
chr8_E32U	chr8	128210009	128209759-128210009
chr8_E32D	chr8	128210009	128210009-128210259
chr8_E33U	chr8	128212245	128211995-128212245
chr8_E33D	chr8	128212245	128212245-128212495
chr8_E34U	chr8	128213061	128212811-128213061
chr8_E34D	chr8	128213061	128213061-128213311

chr8_E35U	chr8	128218632	128218382-128218632
chr8_E35D	chr8	128218632	128218632-128218882
chr8_E36U	chr8	128218932	128218682-128218932
chr8_E36D	chr8	128218932	128218932-128219182
chr8_E37U	chr8	128219174	128218924-128219174
chr8_E37D	chr8	128219174	128219174-128219424
chr8_E38U	chr8	128219906	128219656-128219906
chr8_E38D	chr8	128219906	128219906-128220156
chr8_E39U	chr8	128220095	128219845-128220095
chr8_E39D	chr8	128220095	128220095-128220345
chr8_E40U	chr8	128222727	128222477-128222727
chr8_E40D	chr8	128222727	128222727-128222977
chr8_E41U	chr8	128226201	128225951-128226201
chr8_E41D	chr8	128226201	128226201-128226451
chr8_E42U	chr8	128226307	128226057-128226307
chr8_E42D	chr8	128226307	128226307-128226557
chr8_E43U	chr8	128236071	128235821-128236071
chr8_E43D	chr8	128236071	128236071-128236321
chr8_E44U	chr8	128238907	128238657-128238907
chr8_E44D	chr8	128238907	128238907-128239157
chr8_E45U	chr8	128242519	128242269-128242519
chr8_E45D	chr8	128242519	128242519-128242769
chr8_E46U	chr8	128244004	128243754-128244004
chr8_E46D	chr8	128244004	128244004-128244254
chr8_E47U	chr8	128244486	128244236-128244486
chr8_E47D	chr8	128244486	128244486-128244736
chr8_E48U	chr8	128245792	128245542-128245792
chr8_E48D	chr8	128245792	128245792-128246042
chr8_E49U	chr8	128247176	128246926-128247176
chr8_E49D	chr8	128247176	128247176-128247426
chr8_E50U	chr8	128256336	128256086-128256336
chr8_E50D	chr8	128256336	128256336-128256586
chr8_E51U	chr8	128259713	128259463-128259713
chr8_E51D	chr8	128259713	128259713-128259963
chr8_E52U	chr8	128262818	128262568-128262818
chr8_E52D	chr8	128262818	128262818-128263068
chr8_E53U	chr8	128264277	128264027-128264277
chr8_E53D	chr8	128264277	128264277-128264527
chr8_E54U	chr8	128267176	128266926-128267176
chr8_E54D	chr8	128267176	128267176-128267426
chr8_E55U	chr8	128272843	128272593-128272843
chr8_E55D	chr8	128272843	128272843-128273093
chr8_E56U	chr8	128273239	128272989-128273239

chr8_E56D	chr8	128273239	128273239-128273489
chr8_E57U	chr8	128277169	128276919-128277169
chr8_E57D	chr8	128277169	128277169-128277419
chr8_E58U	chr8	128277386	128277136-128277386
chr8_E58D	chr8	128277386	128277386-128277636
chr8_E59U	chr8	128285865	128285615-128285865
chr8_E59D	chr8	128285865	128285865-128286115
chr8_E60U	chr8	128286221	128285971-128286221
chr8_E60D	chr8	128286221	128286221-128286471
chr8_E61U	chr8	128286578	128286328-128286578
chr8_E61D	chr8	128286578	128286578-128286828
chr8_E62U	chr8	128291605	128291355-128291605
chr8_E62D	chr8	128291605	128291605-128291855
chr8_E63U	chr8	128292140	128291890-128292140
chr8_E63D	chr8	128292140	128292140-128292390
chr8_E64U	chr8	128292999	128292749-128292999
chr8_E64D	chr8	128292999	128292999-128293249
chr8_E65U	chr8	128300231	128299981-128300231
chr8_E65D	chr8	128300231	128300231-128300481
chr8_E66U	chr8	128301697	128301447-128301697
chr8_E66D	chr8	128301697	128301697-128301947
chr8_E67U	chr8	128302776	128302526-128302776
chr8_E67D	chr8	128302776	128302776-128303026
chr8_E68U	chr8	128303983	128303733-128303983
chr8_E68D	chr8	128303983	128303983-128304233
chr8_E69U	chr8	128304296	128304046-128304296
chr8_E69D	chr8	128304296	128304296-128304546
chr8_E70U	chr8	128310266	128310016-128310266
chr8_E70D	chr8	128310266	128310266-128310516
chr8_E71U	chr8	128313979	128313729-128313979
chr8_E71D	chr8	128313979	128313979-128314229
chr8_E72U	chr8	128322127	128321877-128322127
chr8_E72D	chr8	128322127	128322127-128322377
chr8_E73U	chr8	128327244	128326994-128327244
chr8_E73D	chr8	128327244	128327244-128327494
chr8_E74U	chr8	128327764	128327514-128327764
chr8_E74D	chr8	128327764	128327764-128328014
chr8_E75U	chr8	128337132	128336882-128337132
chr8_E75D	chr8	128337132	128337132-128337382
chr8_E76U	chr8	128346166	128345916-128346166
chr8_E76D	chr8	128346166	128346166-128346416
chr8_E77U	chr8	128349689	128349439-128349689
chr8_E77D	chr8	128349689	128349689-128349939

chr8_E78U	chr8	128353277	128353027-128353277
chr8_E78D	chr8	128353277	128353277-128353527
chr8_E79U	chr8	128355485	128355235-128355485
chr8_E79D	chr8	128355485	128355485-128355735
chr8_E80U	chr8	128363305	128363055-128363305
chr8_E80D	chr8	128363305	128363305-128363555
chr8_E81U	chr8	128363660	128363410-128363660
chr8_E81D	chr8	128363660	128363660-128363910
chr8_E82U	chr8	128366238	128365988-128366238
chr8_E82D	chr8	128366238	128366238-128366488
chr8_E83U	chr8	128370870	128370620-128370870
chr8_E83D	chr8	128370870	128370870-128371120
chr8_E84U	chr8	128375845	128375595-128375845
chr8_E84D	chr8	128375845	128375845-128376095
chr8_E85U	chr8	128378575	128378325-128378575
chr8_E85D	chr8	128378575	128378575-128378825
chr8_E86U	chr8	128380889	128380639-128380889
chr8_E86D	chr8	128380889	128380889-128381139
chr8_E87U	chr8	128383742	128383492-128383742
chr8_E87D	chr8	128383742	128383742-128383992
chr8_E88U	chr8	128383751	128383501-128383751
chr8_E88D	chr8	128383751	128383751-128384001
chr8_E89U	chr8	128385200	128384950-128385200
chr8_E89D	chr8	128385200	128385200-128385450
chr8_E90U	chr8	128388244	128387994-128388244
chr8_E90D	chr8	128388244	128388244-128388494
chr8_E91U	chr8	128391894	128391644-128391894
chr8_E91D	chr8	128391894	128391894-128392144
chr8_E92U	chr8	128401541	128401291-128401541
chr8_E92D	chr8	128401541	128401541-128401791
chr8_E93U	chr8	128421240	128420990-128421240
chr8_E93D	chr8	128421240	128421240-128421490
chr8_E94U	chr8	128421540	128421290-128421540
chr8_E94D	chr8	128421540	128421540-128421790
chr8_E95U	chr8	128423061	128422811-128423061
chr8_E95D	chr8	128423061	128423061-128423311
chr8_E96U	chr8	128438740	128438490-128438740
chr8_E96D	chr8	128438740	128438740-128438990
chr8_E97U	chr8	128438789	128438539-128438789
chr8_E97D	chr8	128438789	128438789-128439039
chr8_E98U	chr8	128442095	128441845-128442095
chr8_E98D	chr8	128442095	128442095-128442345
chr8_E99U	chr8	128447517	128447267-128447517

chr8_E99D	chr8	128447517	128447517-128447767
chr8_E100U	chr8	128448560	128448310-128448560
chr8_E100D	chr8	128448560	128448560-128448810
chr8_E101U	chr8	128450698	128450448-128450698
chr8_E101D	chr8	128450698	128450698-128450948
chr8_E102U	chr8	128458371	128458121-128458371
chr8_E102D	chr8	128458371	128458371-128458621
chr8_E103U	chr8	128458451	128458201-128458451
chr8_E103D	chr8	128458451	128458451-128458701
chr8_E104U	chr8	128459569	128459319-128459569
chr8_E104D	chr8	128459569	128459569-128459819
chr8_E105U	chr8	128460403	128460153-128460403
chr8_E105D	chr8	128460403	128460403-128460653
chr8_E106U	chr8	128461598	128461348-128461598
chr8_E106D	chr8	128461598	128461598-128461848
chr8_E107U	chr8	128464161	128463911-128464161
chr8_E107D	chr8	128464161	128464161-128464411
chr8_E108U	chr8	128476827	128476577-128476827
chr8_E108D	chr8	128476827	128476827-128477077
chr8_E109U	chr8	128478928	128478678-128478928
chr8_E109D	chr8	128478928	128478928-128479178
chr8_E110U	chr8	128478942	128478692-128478942
chr8_E110D	chr8	128478942	128478942-128479192
chr8_E111U	chr8	128482067	128481817-128482067
chr8_E111D	chr8	128482067	128482067-128482317
chr8_E112U	chr8	128485305	128485055-128485305
chr8_E112D	chr8	128485305	128485305-128485555
chr8_E113U	chr8	128489041	128488791-128489041
chr8_E113D	chr8	128489041	128489041-128489291
chr8_E114U	chr8	128490983	128490733-128490983
chr8_E114D	chr8	128490983	128490983-128491233
chr8_E115U	chr8	128491988	128491738-128491988
chr8_E115D	chr8	128491988	128491988-128492238
chr8_E116U	chr8	128492229	128491979-128492229
chr8_E116D	chr8	128492229	128492229-128492479
chr8_E117U	chr8	128496205	128495955-128496205
chr8_E117D	chr8	128496205	128496205-128496455
chr8_E118U	chr8	128499380	128499130-128499380
chr8_E118D	chr8	128499380	128499380-128499630
chr8_E119U	chr8	128500218	128499968-128500218
chr8_E119D	chr8	128500218	128500218-128500468
chr8_E120U	chr8	128500865	128500615-128500865
chr8_E120D	chr8	128500865	128500865-128501115

chr8_E121U	chr8	128502909	128502659-128502909
chr8_E121D	chr8	128502909	128502909-128503159
chr8_E122U	chr8	128506239	128505989-128506239
chr8_E122D	chr8	128506239	128506239-128506489
chr8_E123U	chr8	128508276	128508026-128508276
chr8_E123D	chr8	128508276	128508276-128508526
chr8_E124U	chr8	128510027	128509777-128510027
chr8_E124D	chr8	128510027	128510027-128510277
chr8_E125U	chr8	128511052	128510802-128511052
chr8_E125D	chr8	128511052	128511052-128511302
chr8_E126U	chr8	128513845	128513595-128513845
chr8_E126D	chr8	128513845	128513845-128514095
chr8_E127U	chr8	128517428	128517178-128517428
chr8_E127D	chr8	128517428	128517428-128517678
chr8_E128U	chr8	128520894	128520644-128520894
chr8_E128D	chr8	128520894	128520894-128521144
chr8_E129U	chr8	128521424	128521174-128521424
chr8_E129D	chr8	128521424	128521424-128521674
chr8_E130U	chr8	128537496	128537246-128537496
chr8_E130D	chr8	128537496	128537496-128537746
chr8_E131U	chr8	128538718	128538468-128538718
chr8_E131D	chr8	128538718	128538718-128538968
chr8_E132U	chr8	128546634	128546384-128546634
chr8_E132D	chr8	128546634	128546634-128546884
chr8_E133U	chr8	128549432	128549182-128549432
chr8_E133D	chr8	128549432	128549432-128549682
chr8_E134U	chr8	128551222	128550972-128551222
chr8_E134D	chr8	128551222	128551222-128551472
chr8_E135U	chr8	128551676	128551426-128551676
chr8_E135D	chr8	128551676	128551676-128551926
chr10_E1U	chr10	51513346	51513096-51513346
chr10_E1D	chr10	51513346	51513346-51513596
chr10_E2U	chr10	51514825	51514575-51514825
chr10_E2D	chr10	51514825	51514825-51515075
chr10_E3U	chr10	51515499	51515249-51515499
chr10_E3D	chr10	51515499	51515499-51515749
chr10_E4U	chr10	51521264	51521014-51521264
chr10_E4D	chr10	51521264	51521264-51521514
chr10_E5U	chr10	51522097	51521847-51522097
chr10_E5D	chr10	51522097	51522097-51522347
chr10_E6U	chr10	51525116	51524866-51525116
chr10_E6D	chr10	51525116	51525116-51525366
chr10_E7U	chr10	51527984	51527734-51527984

chr10_E7D	chr10	51527984	51527984-51528234
chr10_E8U	chr10	51530280	51530030-51530280
chr10_E8D	chr10	51530280	51530280-51530530
chr10_E9U	chr10	51533001	51532751-51533001
chr10_E9D	chr10	51533001	51533001-51533251
chr10_E10U	chr10	51547645	51547395-51547645
chr10_E10D	chr10	51547645	51547645-51547895
chr11_E1U	chr11	68978565	68978315-68978565
chr11_E1D	chr11	68978565	68978565-68978815
chr11_E2U	chr11	68981087	68980837-68981087
chr11_E2D	chr11	68981087	68981087-68981337
chr11_E3U	chr11	68982776	68982526-68982776
chr11_E3D	chr11	68982776	68982776-68983026
chr11_E4U	chr11	68987665	68987415-68987665
chr11_E4D	chr11	68987665	68987665-68987915
chr11_E5U	chr11	68987859	68987609-68987859
chr11_E5D	chr11	68987859	68987859-68988109
chr11_E6U	chr11	68988122	68987872-68988122
chr11_E6D	chr11	68988122	68988122-68988372
chr11_E7U	chr11	68990851	68990601-68990851
chr11_E7D	chr11	68990851	68990851-68991101
chr11_E8U	chr11	68992301	68992051-68992301
chr11_E8D	chr11	68992301	68992301-68992551
chr11_E9U	chr11	68995786	68995536-68995786
chr11_E9D	chr11	68995786	68995786-68996036
chr11_E10U	chr11	68996149	68995899-68996149
chr11_E10D	chr11	68996149	68996149-68996399
chr11_E11U	chr11	68998040	68997790-68998040
chr11_E11D	chr11	68998040	68998040-68998290
chr11_E12U	chr11	69003234	69002984-69003234
chr11_E12D	chr11	69003234	69003234-69003484
chr11_E13U	chr11	69003826	69003576-69003826
chr11_E13D	chr11	69003826	69003826-69004076
chr11_E14U	chr11	69010038	69009788-69010038
chr11_E14D	chr11	69010038	69010038-69010288
chr11_E15U	chr11	69011179	69010929-69011179
chr11_E15D	chr11	69011179	69011179-69011429
chr11_E16U	chr11	69015236	69014986-69015236
chr11_E16D	chr11	69015236	69015236-69015486
chr11_E17U	chr11	69016634	69016384-69016634
chr11_E17D	chr11	69016634	69016634-69016884
chr11_E18U	chr11	69019607	69019357-69019607
chr11_E18D	chr11	69019607	69019607-69019857

chr11_E19U	chr11	69024388	69024138-69024388
chr11_E19D	chr11	69024388	69024388-69024638
chr11_E20U	chr11	69031326	69031076-69031326
chr11_E20D	chr11	69031326	69031326-69031576

chr: chromosome; E: EcoRI digestion site; U: upstream; D: downstream; A: the first region on the chr.; B: the second region on the same chr.

Supplementary Table S3. Correlation between LNCaP and other l libraries

Cell lines	LNCaP-1	
	Cutoff 1	Cutoff 3
LNCaP-2	0.90	0.93
DU-145	0.79	0.83
BPH-1	0.55	0.57
RWPE-1	0.45	0.43
LCL-29092	0.82	0.85
LCL-32370	0.76	0.8

Supplementary Table S4. Target genes and their potential regulatory SNPs

Chr.	Genes	Expression Level (RPKM)	Risk SNP	eQTL SNP	eQTL P-value	Peak fragments
1q21.3	UBE2Q1	13.27	rs1218582	rs1218582	4.53E-01	No
1q21.3	KCNN3	1.23	rs1218582	rs1218582	4.80E-01	No
1q32.1	PPP1R15B	15.95	rs4245739	rs4245739	5.29E-01	No
1q32.1	PIK3C2B	2.74	rs4245739	rs4245739	9.34E-01	No
1q32.1	NFASC	4.34	rs4245739	rs4245739	1.35E-01	BE15-16
2p24.1	C2orf43	3.97	rs13385191	rs13394027	**2.25E-27**	AE3-4
2p24.1	HS1BP3	11.78	rs13385191	rs10170771	3.94E-01	AE3-6
2p11.2	CAPG	33.90	rs10187424	rs1078004	**3.48E-03**	BE7-8
2p11.2	CAPG	33.90	rs2028898	rs1446669	**3.93E-03**	BE5-6
2p11.2	CAPG	33.90	rs2028898	rs699664	**4.37E-03**	BE8-9
2p11.2	USP39	13.09	rs2028898	rs1446669	8.44E-01	BE5-6
2p11.2	USP39	13.09	rs2028898	rs699664	9.52E-01	BE8-9
5p15.33	TRIP13	0.40	rs2242652	rs7725218	2.40E-02	AE2-3
6q22.1	GPRC6A	0.07	rs339331	rs339331	8.76E-01	No
6q22.1	GPRC6A	0.07	rs339331	rs1321366	9.36E-01	No
6q22.1	RFX6	0.07	rs339331	rs339331	2.49E-01	No
6q22.1	RFX6	0.07	rs339331	rs1321366	2.83E-01	No
8q24.21	PVT1	0.92	rs16901979	rs16901966	8.04E-02	E6-7
8q24.21	MYC	21.79	rs16901979	rs16901966	3.71E-01	E6-7
8q24.21	PCAT1	0.16	rs16901979	rs16901966	4.48E-01	E6-7
8q24.21	PVT1	0.92	rs10808556	rs10808556	**8.38E-04**	E92-93
8q24.21	MYC	21.79	rs10808556	rs10808556	2.90E-01	E92-93
8q24.21	PCAT1	0.16	rs10808556	rs10808556	4.88E-01	E92-93
8q24.21	PVT1	0.92	rs6983267	rs6983267	**1.75E-03**	E92-93
8q24.21	MYC	21.79	rs6983267	rs6983267	5.89E-01	E92-93
8q24.21	PCAT1	0.16	rs6983267	rs6983267	9.16E-02	E92-93
8q24.21	PCAT1	0.16	rs10086908	rs10086908	**9.38E-03**	E92-93
8q24.21	MYC	21.79	rs10086908	rs10086908	4.61E-01	E92-93
8q24.21	PVT1	0.92	rs10086908	rs10086908	5.93E-01	E92-93
8q24.21	CASC8/LOC727677	0.01	rs6983267	rs6983267	9.38E-03	E92-93
10q11.23	AGAP7	3.58	rs10993994	rs10993994	**4.06E-07**	E10D
10q11.23	AGAP7	3.58	rs10993994	rs4631830	**8.02E-05**	E10U

Supplementary Table S5. Differential gene expression between normal and tumor tissues from TCGA

Genes	p-value	Mean Tumor	Mean Normal	Fold Change
GPRC6A	6.64E-22	0.66	2.25	-3.01
TRIP13	9.25E-22	6.09	4.74	2.55
CAPG	4.88E-19	9.35	10.77	-2.67
PVT1	1.22E-17	6.83	5.77	2.08
C2orf43	3.56E-10	7.12	8.40	-2.43
MYC	1.55E-09	11.01	10.17	1.80
UBE2Q1	3.90E-09	10.63	10.42	1.16
NFASC	7.79E-06	8.21	8.93	-1.65
KCNN3	2.72E-05	3.84	4.60	-1.69
CASC8	1.27E-04	0.55	0.05	1.42
PIK3C2B	2.51E-04	8.86	9.17	-1.24
RFX6	9.36E-04	3.60	2.67	1.92
AGAP7	7.18E-03	6.23	5.77	1.38
PPP1R15B	9.23E-03	10.72	10.90	-1.13
USP39	0.47	9.95	9.98	-1.02
HS1BP3	0.87	10.16	10.15	1.01
PCAT1	no data	no data	no data	no data

Supplementary Table S6. Sequences of 3C primers and TaqMan probes used to validate the interaction between 2p11.2 region and CAPG

Primers or probes for 3C-qPCR

Name	Sequences
chr2_ E9U_anchor	5'-ACAATGCCTCTGGCTAGTCC-3'
chr2_ E9U_probe	5'-TGTAGCCTGGGAGGAAACAGGAACCACAGT-3'
chr2_T3_ E9U_RC	5'-CCATTTGGAAATTAGGTGGATTCC-3'
chr2_T4 _E9U_RC	5'-TGGAATGGGGCTTGATCACC-3'
chr2_T6 _E9U_RC	5'-CAACCAGAACAGTGACATGCTG-3'
chr2_T7 _E9U_RC	5'-GAGTTTCTAGTAAGTCCACTTCCAG-3'
chr2_T8 _E9U_RC	5'-CGGAGGCACCCTAAGGAGAC-3'
chr2_T9 _E9U_RC	5'-CATTGGCCCACCTCAGCCAC-3'
chr2_T10 _E9U_RC	5'-GCTCTACTGTGGCTGTCAGG-3'
chr2_T11 _E9U_RC	5'-GACACCAGGCCTCCAGAGTA-3'
chr2_T12_ E9U_RC	5'-CTGCCACACTGGCCTCAGAC-3'

Primers for 3C template controls

Name	Sequences
chr2_C_E7D_F	5'-TCAGGTAGAGAAACAAATACAAAG-3'
chr2_C_E7D_R	5'-TGGAGATAGATAATGGATGAGAAA -3'
chr2_C_NC1_F	5'-GGGAAAACACAAAAGAAGAGAAAC-3'
chr2_C_NC1_R	5'-ATAGCATTGAAGAAAATACCGCAC-3'
chr2_C_NC2_F	5'-CTTAAGGGAAGCAACATACTGGAG-3'
chr2_C_NC2_R	5'-TAGGAACTTTTTGAAATAGGGCAA-3'
chr2_C_T3_F	5'-TCAAGGGCAAATGGGCTGGAGT-3'
chr2_C_T3_R	5'-GGGCATGATGGGCTGGGAAG-3'
chr2_C_T4_F	5'-AGGAATCCTGTTAGCTCAGTCTAGCCAGAA-3'
chr2_C_T4_R	5'-GAGCTTCTCAAACACTGACATCCAGG-3'
chr2_C_T5_F	5'-CCACCCTCGAAACCAAACTGAA-3'
chr2_C_T5_R	5'-GAAGACGAACCTACGAAGCAGA-3'
chr2_C_T6_F	5'-ATTCAGGATGCTTCTAGCCA-3'
chr2_C_T6_R	5'-GACTCCCACTACATGGCTCC-3'
chr2_C_T7_F	5'-TCTGTGTTGACTGTTATTGTGT-3'
chr2_C_T7_R	5'-GTTCTACTGGGTCTATTCTTTG-3'
chr2_C_T8_F	5'-AGGCCTGCCAAGTCAGTTGG-3'
chr2_C_T8_R	5'-CCATGGGTTTGACCAGTCCT-3'
chr2_C_T9_F	5'-CAACCCTGAACCAATTCCTAGA-3'
chr2_C_T9_R	5'-GGAGTTTGTCCTTCCTCATCCC-3'
chr2_C_T10_F	5'-AGGCTCAGTTCTAGGGTGGA-3'

chr2_C_T10_R	5'-TACCTGACAGAGGCTGCCAT-3'
chr2_C_T11_F	5'-TAAATATTAATGGCCGGAAGTG-3'
chr2_C_T11_R	5'-GGAGTGTGAACAAGGACAGAGA-3'
chr2_C_T12_F	5'-CAGCAAACCCTGTTGGCTCTACC-3'
chr2_C_T12_R	5'-TTGCCCCTATCCAGCGGGAA-3'
chr2_C_E6_F	5'-TGATGCCCACCTTCAGCAGG-3'
chr2_C_E6_R	5'-AAAGTGCCATTACGGCGTAGTTCTG-3'
chr2_C_E8_F	5'-TGCCAGGTCTTGCTCCAGTG-3'
chr2_C_E8_R	5'-TGGGAGTGAGTTCACTCTGC-3'
chr2_C_E9_F	5'-ACAACATACTAACTGAGCCCA-3'
chr2_C_E9_R	5'-CAAACAACCTAGAGGAGTGTCTT-3'

Primers for site directed mutagenesis for SNP rs4631830

Name	Sequences
rs4631830CtoT-F	5'-GTTTCTCAGCGGCTAATCTGGAACATGTTTGC-3'
rs4631830CtoT-R	5'-GCAAACATGTTCCAGATTAGCCGCTGAGAAAC-3'

Chr: chromosome; E: EcoRI digestion site; C: control; F: forward primer; R: reverse primer; U: upstream; D: downstream; NC:negative control
